# Unveiling the suitable habitats and future conservation strategies of *Tridacna maxima* in the Indo‐Pacific core area based on species distribution model

**DOI:** 10.1002/ece3.70187

**Published:** 2024-09-04

**Authors:** Shenghao Liu, Tingting Li, Bailin Cong, Leyu Yang, Zhaohui Zhang, Linlin Zhao

**Affiliations:** ^1^ Marine Ecology Research Center, Observation and Research Station of Bohai Strait Eco‐Corridor First Institute of Oceanography, Ministry of Natural Resources Qingdao China; ^2^ Laboratory for Marine Ecology and Environmental Science Qingdao Marine Science and Technology Center Qingdao China; ^3^ School of Advanced Manufacturing Fuzhou University Jinjiang China

**Keywords:** climate change, conservation, habitat suitability, niche difference, species distribution models, *Tridacna maxima*

## Abstract

Climate change is exerting unprecedented impacts on marine habitats, and many sessile invertebrate species, such as the endangered giant clam *Tridacna maxima*, are particularly sensitive to climate driven changes in their environment. Understanding its spatial distribution and conservation requirements is of crucial significance in formulating effective protection strategies. However, the species has been extensively harvested and depleted in many regions, leading to its listing as endangered species by the International Union for Conservation of Nature (IUCN). While marine protected areas (MPAs) are considered effective conservation tools, it remains uncertain whether existing MPAs adequately protect these vulnerable giant clams. To enhance the management and conservation of this species, we employed a Species Distribution Models (SDMs) approach, integrating occurrence records of *T. maxima* with environmental variables, to predict its potential distribution based on habitat suitability and capture spatiotemporal changes. Based on geographical and genetic variations, the *T. maxima* in the Indo‐Pacific core region is primarily divided into two populations: the East Indian Ocean‐South China Sea population (EIOS) and the West Pacific‐Indonesia population (WPI). We first quantified realized niche to reveal significant differences in ecological niche space among different populations. Subsequently, SDMs were constructed at both species and population levels, demonstrating that population‐level SDMs provide more reliable predictions of population distributions due to differential responses to climatic predictor variables. Finally, we conducted an assessment to identify conservation gaps for *T. maxima* beyond the existing MPAs and proposed recommendations for future establishment of MPAs within the current framework. Based on these findings, appropriate conservation policies have been proposed to effectively protect the habitat of different geographical populations of *T. maxima*. Additionally, spatiotemporal predictions of habitat suitability provide crucial information for developing adaptive management strategies for *T. maxima* in response to climate change, including designing new protected areas and adjusting the location and extent of existing protected areas based on their geographical distribution.

## Abstract 2

We employed a Species Distribution Models (SDMs) approach, integrating occurrence records of *Tridacna maxima* with environmental variables, to predict its potential distribution based on habitat suitability and capture spatiotemporal changes. In addition, we conducted an assessment to identify conservation gaps beyond the existing MPAs and proposed recommendations for the future establishment of MPAs within the current framework.

## Graphical abstract

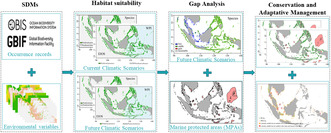

## 1 INTRODUCTION

Environmental factors constrain the growth, development, and geographic distribution of species. The longevity and reproductive success of marine organisms hinge on their adaptations to localized ecological niches. Unfortunately, climate change and human activities have triggered a series of alterations within the marine environment, including elevated water temperatures, diminished primary productivity, ocean acidification, and hypoxia (Cheung et al., 2013; Lumpkin et al., 2020). These alterations have the potential to surpass physiological and ecological thresholds, leading to habitat loss, reduced abundance, and even the extinction of numerous species (Duncan et al., 2023; Penn & Deutsch, 2022). Compared with terrestrial communities, marine communities are more susceptible to environmental changes induced by climate change (Sorte et al., 2010). Mollusks are widely distributed and important members of marine ecosystems and play a crucial role in maintaining biodiversity and regulating ecological processes. As benthic organisms, mollusks are highly sensitive to environmental changes and can provide important indicators of the health and stability of marine ecosystems (Moraitis et al., 2018). Therefore, comprehensive and accurate understanding of the current distribution of mollusks, as well as reliable predictions regarding their responses to future climate change, is crucial for the development of effective resource management and species conservation strategies.

Species Distribution Models (SDMs) are currently valuable tools for predicting potential species distribution (Anibaba et al., 2022; Guisan et al., 2017). The underlying principle of SDMs involves using existing species distribution data and environmental variables to establish ecological relationships based on the species niches. This approach investigates the relationships between environmental characteristics within known distribution areas and those within potential distribution areas to predict the potential range of a species (Araújo et al., 2019). It allows for the prediction of current and future species distribution under varying climatic conditions (Booth et al., 2014). Traditionally, SDMs have been constructed based on the “ecological niche conservatism” hypothesis, which suggests that individuals of the same species have similar ecological niche spaces and exhibit consistent responses to climate change within their range (Smith et al., 2019). However, local adaptation and intraspecific variation can affect a species' response to environmental change (Li et al., 2022). Therefore, SDMs that only consider the species level, while ignoring intraspecific variation, often overpredict a species' future distribution (Hu et al., 2021; Pack et al., 2022). Consequently, the incorporation of intraspecific genetic differences into the SDM of *T. maxima* holds promise for improving prediction accuracy and precision. Moreover, it facilitates a more nuanced understanding of the species' ecological requirements and unveils the complexity of population dynamics and distribution patterns. (Hu et al., 2021).

**FIGURE 1:**
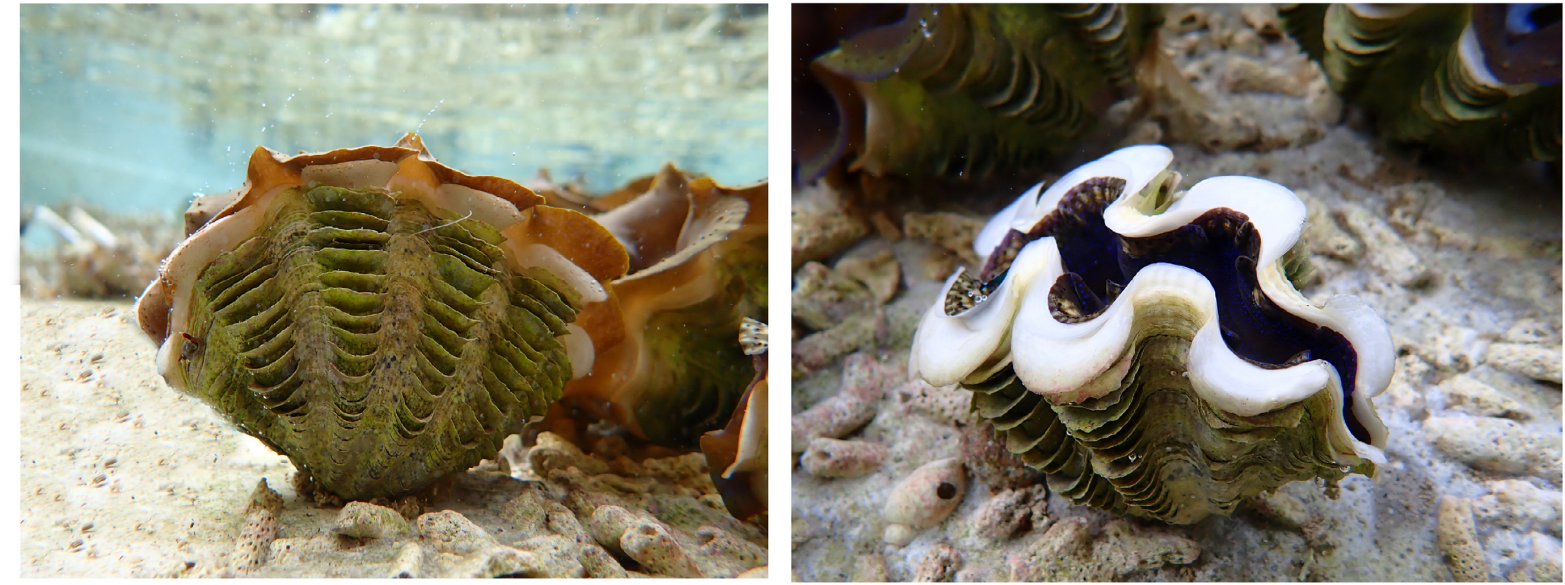
The photos of *Tridacna maxima*.

*Tridacna maxima* (Figure 1), a significant coral reef inhabitant, plays a key role in the Indian and Pacific Oceans. They provide habitat, breeding grounds, and shelter for other reef organisms, thus playing a crucial role in marine environments, particularly coral reef ecosystems. Over the past two decades, *Tridacna* populations have suffered substantial damage due to human activities and global climate changes, leading to critical endangerment for most species (Andréfouët et al., 2013; Neo et al., 2015). In response to these impacts, *T. maxima* has been listed as an endangered species designation by the “China Red List” and listed as a Class II protected wild animal designation by the “National Key Protected Wildlife List”. Genetic studies on the *T. maxima* population have revealed that within the Indo‐Pacific core region, two main populations exist: the Eastern Indian Ocean‐South China Sea (EIOS) and the Western Pacific‐Indonesia (WPI) populations (Hui et al., 2016; Nuryanto & Kochzius, 2009). Although the degree of distribution overlap and genetic exchange between populations remains uncertain, these two populations have inhabited distinct ecological environments throughout their extensive evolutionary history, potentially leading to local adaptations.

Marine protected areas (MPAs) have become a widespread conservation strategy employed to protect and manage marine biodiversity as well as support sustainable use of ocean resources (Roberts et al., 2018). To date, despite a series of efforts have been made to conserve *Tridacna* species, there is limited information on the protected status and distribution of *T. maxima* in the study region. To gain a profound understanding of the existing gaps in protection coverage, a gap analysis is crucial, leveraging data on current MPAs and suitable habitats. In this study, we favor species models over population models, as while population models may yield precision in localized studies, their application across vast and diverse regions is limited. As Margules and Pressey (2000) observed, species‐level data offer distinct advantages in systematic conservation planning, boasting high data availability, computational efficiency, and practicality, especially when resources are scarce. Moreover, species‐centric conservation strategies are more comprehensible and implementable, safeguarding entire species rather than isolated populations, which is paramount in addressing future climate and ecological changes. Therefore, our utilization of species models for gap analysis aims to provide more scientific and actionable guidance for the conservation management and planning of endangered species, both presently and in the future.

In this study, which focused on the Indo‐Pacific Core Area, we first quantified realized niche and compared ecological niche space differences among different populations. Subsequently, we constructed SDMs at both the species and population levels to predict the potential distribution of *T. maxima*, and predicted the potential distribution under future climate scenarios. Lastly, we conducted a gap analysis to identify conservation gaps for *T. maxima* beyond the MPAs and proposed recommendations for future establishment of the existing MPAs. The following are some noteworthy contributions made by this study: (1) quantifying the spatial differences in ecological niche occupied by the two populations; (2) determining the important environmental factors affecting *T. maxima* distribution; (3) creating maps of habitat suitability for *T. maxima* under current and future climate scenarios; and (4) assessing the substantial gaps in *T. maxima* protection in the current MPAs' areas. These research findings can provide important insights for the development of adaptive management strategies, including the design of new protected areas and adjustment of existing protected area locations and extents based on the geographical distribution of *T. maxima* in current and future scenarios, thus offering valuable references for addressing marine conservation planning issues. Furthermore, this study also offers guidance for investigating the potential distribution of other protected species under future climate change scenarios.

## 2 MATERIALS AND METHODS

### 2.1 Study area and species occurrence data collection

*Tridacna maxima* is mainly distributed in the Indian Ocean and the Western Pacific. The Indo‐West Pacific region, centered around the Indo‐Malay Archipelago, exhibits the highest species diversity in shallow waters of the ocean. Genetic studies of the *T. maxima* have revealed the existence of two primary populations in the Indo‐Pacific core region: the East Indian Ocean‐South China Sea (EIOS) and the Western Pacific‐Indonesian (WPI) populations (Hui et al., 2016; Nuryanto & Kochzius, 2009). Although the precise boundary between these two populations remains undefined, a comprehensive analysis of current research suggests that their lineages follow the “Wallace's Line,” a classical biogeographical exemplar in evolutionary biology. To further delineate this boundary, we have consulted relevant literature (Tian et al., 2023) and integrated oceanographic and geophysical parameters between the EIOS and WPI regions. Utilizing Geographic Information Systems (GIS) for spatial analysis, we have identified the boundary between EIOS and WPI. The EIOS population primarily encompasses areas west of the Indonesian Throughflow (ITF) and Makassar Strait, while the WPI population comprises the regions eastward of these geographical features. While molecular genetic techniques were not employed, our approach still offers a reasonable population delineation, contributing to a deeper understanding of the genetic structure and distribution patterns of the *T. maxima*.

**FIGURE 2:**
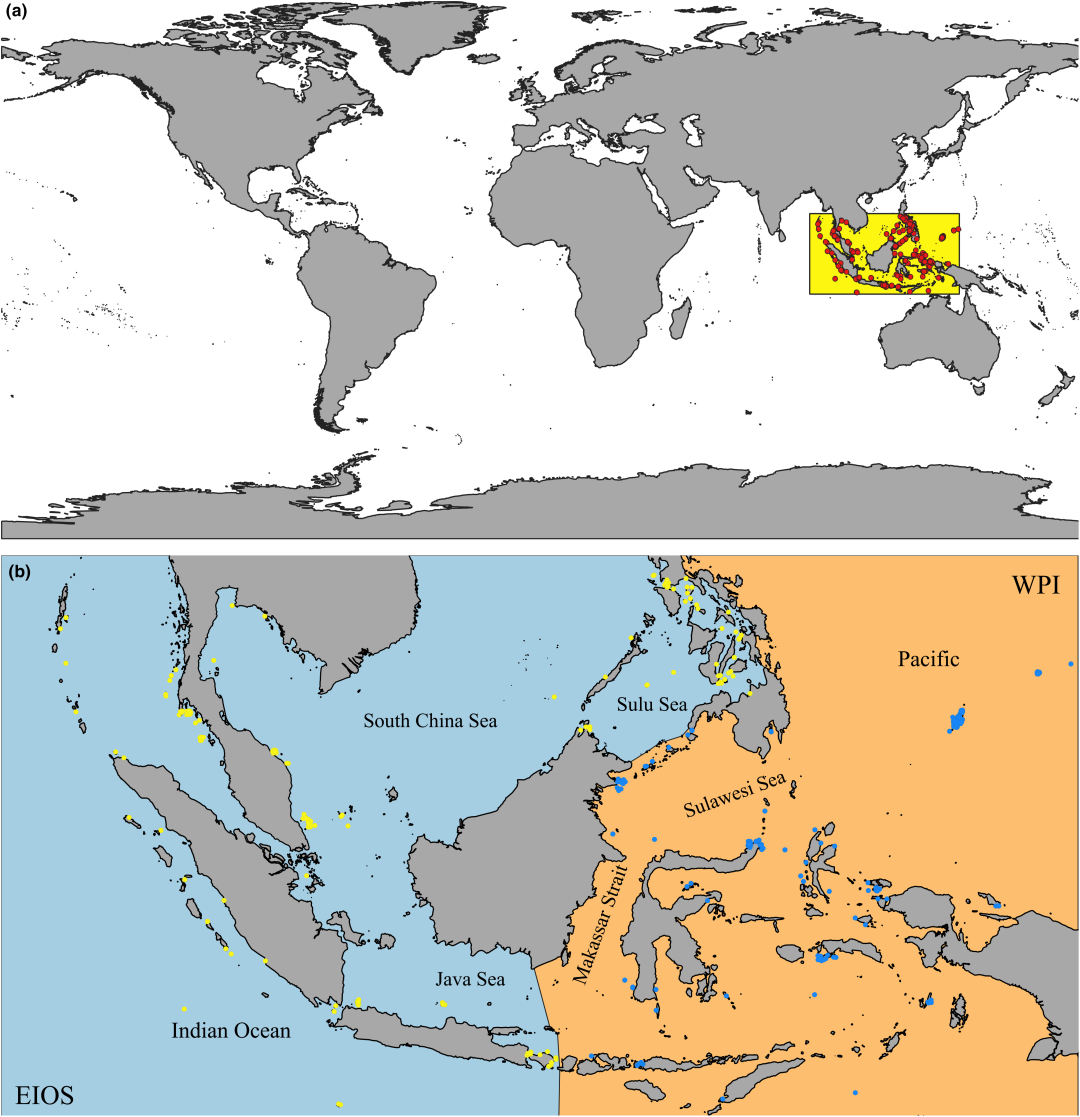
Map of the study area and populations of *Tridacna maxima*. (a) Map showing the study area and occurrence records of Tridacna maxima. (b) Locations of two populations of Tridacna maxima: yellow and blue dots indicate the East Indian Ocean‐South China Sea population (EIOS) and the West Pacific‐Indonesian population (WPI), respectively.

Our study focuses on a limited area of 90–140° E, 11° S–15° N, based on the known distribution ranges of the two populations. Occurrence records of *T. maxima* were collected from online public databases such as the Global Biodiversity Information Facility (GBIF; http://www.gbif.org/, accessed on January 13, 2023), and the Ocean Biogeographic Information System (OBIS; https://obis.org/, accessed on January 13, 2023). To minimize sampling bias, we employed the R software package *spThin* for spatial refinement of the distribution data (Aiello‐Lammens et al., 2015). Each 5 × 5 arc‐minutes grid (i.e., approximately 9.2 km × 9.2 km) was assigned one occurrence point, consistent with the environmental predictors' resolution. Following this data cleaning procedure, which included removing duplicates, eliminating records with missing information, and filtering out non‐marine data points, we retrieved 213 records within our study area to build the SDM at the species level (hereafter “species model”). Of these, 113 records belonged to the EIOS that was used to construct the SDM at the population level (hereafter “EIOS model”), and 100 records belonged to the WPI that was used to construct the WPI model (Figure 2). The available data reflected the presence of the *T. maxima*, whereas most SDMs require information on species absence (Singer et al., 2017). Given the absence of authentic absence records, we resorted to randomly simulating an equal number of pseudo‐absence records in the environment conditions where the *T. maxima* did not occur to match the presence records using the pseudo‐absences function within the R package MOPA (Barbet‐Massin et al., 2012). The resulting binary presence/absence (0/1) dataset was used as the response variable to construct the SDM.

### 2.2 Environmental predictor variables

Considering the influence of habitat surroundings on the distribution of *T. maxima*, as well as a combination of bioenvironmental relevance and data availability, we identified nine environmental variables that could potentially serve as predictors for the occurrence of *T. maxima* (Table 1). Water temperature, current velocity, salinity, and ocean depth have been proven to be the critical factors limiting the distribution of marine species (Parmesan & Yohe, 2003; Poloczanska et al., 2013). *T. maxima* is omnivorous, usually feeding on phytoplankton, organic debris, and small zooplankton. Dissolved oxygen and light intensity are important factors that affect the distribution of food for *T. maxima* (Klumpp & Lucas, 1994; MingoaLicuanan & Lucas, 1995), thus they were selected as predictive factors. Additionally, considering that *T. maxima* has a certain adaptability to water depth, both too shallow and too deep water may be unfavorable for its survival (Jennings et al., 2013), hence we included water depth as a predictive factor. All the predictor variables were downloaded from the online datasets of Bio‐ORACLE v2.1 dataset (http://www.bio‐oracle.org, accessed on April 6, 2023) (Assis et al., 2018) and Global Marine Environment Datasets (http://gmed.auckland.ac.nz, accessed on April 15, 2023) (Basher et al., 2018) with a spatial resolution of 5 × 5 arc‐minutes (i.e., 9.2 km × 9.2 km; Table 1).

**TABLE 1:** The nine environmental variables selected for this study.

Environment variable	Unit	Spatial resolution	Source	Data for the future
Current velocity	m S^−1^	5 arc min	http://www.bio‐oracle.org	Downloaded from Bio‐oracle
Salinity	PSS	5 arc min	http://www.bio‐oracle.org	Downloaded from Bio‐oracle
Temperature mean	°C	5 arc min	http://www.bio‐oracle.org	Downloaded from Bio‐oracle
Temperature range	°C	5 arc min	http://www.bio‐oracle.org	Downloaded from Bio‐oracle
Depth	m	5 arc min	http://www.bio‐oracle.org	Remain unchanged
Distance to shore	km	5 arc min	http://gmed.auckland.ac.nz	Remain unchanged
Dissolved oxygen	mol m^−3^	5 arc min	http://gmed.auckland.ac.nz	Remain unchanged
Light level at bottom	/	5 arc min	http://www.bio‐oracle.org	Remain unchanged
Phytoplankton	μmol m^−3^	5 arc min	http://www.bio‐oracle.org	Remain unchanged

To reduce the influence of collinearity on the precision of model predictions, we conducted Pearson's correlation factor analysis and the variation inflation factor analysis (VIF) among the nine predictors. Only variables with correlation values <|0.7| and VIF values <10 were retained (Fournier et al., 2017; Zuur et al., 2010). After this process, all nine predictors, including mean current velocity, mean salinity, mean temperature, temperature range, mean dissolved oxygen, light level at bottom, phytoplankton, water depth, and distance to shore were retained for the modeling analysis (Table 1, Figure S1).

Bio‐ORACLE provides future predictive values using three atmospheric–ocean general circulation models (AOGCMs) under four Representative Concentration Pathways (RCPs): RCP 2.6, RCP 4.5, RCP 6.0, and RCP 8.5. We used the average predictive values of three AOGCMs to represent future climate conditions to reduce uncertainty (Assis et al., 2018). In this study, we focused on two RCPs (RCP 2.6 and RCP 8.5), the most optimistic and pessimistic prediction models, for future distribution prediction in the 2050s (2040–2050) and 2100s (2090–2100). Here, future environmental factors, including current velocity, mean salinity, mean temperature, and temperature range, were downloaded from Bio‐ORACLE, while the other factors were assumed to remain unchanged in the future (Table 1) (Zhang et al., 2021).

### 2.3 Estimates of niche spatial differences

To evaluate whether the two populations of *T. maxima* occupy different niche spaces, we employed n‐dimensional hypervolume to quantify the size of their realized niche space (Lê et al., 2008). To this end, we initially conducted principal component analysis (PCA) on the nine selected environmental variables and retained the first four principal components, as they cumulatively explained 81.7% of the total variance (Figure S2). We then used the R package hypervolume (Blonder et al., 2023) to calculate the four‐dimensional hypervolumes of both the EIOS and WPI, based on the retained principal component values corresponding to occurrence records of each population. Finally, we overlapped the hypervolume to estimate the niche spatial differences between these two populations using the R package BAT (Cardoso et al., 2021). The βTotal ranged from 0 to 1, indicating complete overlap to complete separation between the two hypervolumes (Carvalho & Cardoso, 2020). Total niche divergence (βTotal) can be further decomposed into two components: niche shift (spatial replacement between hypervolumes) and niche contraction/expansion (net difference between hypervolumes) (Carvalho & Cardoso, 2020; Mammola & Cardoso, 2020).

### 2.4 Species distribution modeling and prediction

Based on the obtained environmental data and presence/pseudo‐absence records, we developed SDMs to assess the associations between species and the environment. The SDMs were developed from the R package biomod2 (Thuiller et al., 2020) and the package includes 10 modeling algorithms: maximum entropy (Maxent), random forest (RF), surface extent envelope (SRE), multiple adaptive regression splines (MARS), artificial neural network (ANN), flexible discriminant analysis (FDA), classification tree analysis (CTA), generalized boosting model (GBM), generalized linear model (GLM), and generalized additive model (GAM). To evaluate the predictive performance of each model, a five‐fold cross‐validation method with 10 replicates was used (Fu et al., 2021). In this methodology, 80% of the dataset is randomly selected for model calibration and testing, while the remaining 20% is reserved for assessing model predictions (Guisan et al., 2017). We then evaluated the models' accuracy using the true skill statistic (TSS) and area under the ROC curve (AUC) values.

Given the potential diversity in outcomes generated by the 10 individual models, a weighted average ensemble model was constructed to reduce uncertainty and enhance reliability (Buisson et al., 2010; Morato et al., 2020). In this study, a consistent treatment was applied to each model, ensuring equal weighting of predictions. Moreover, the ensemble models utilized the entire dataset, rather than specific subsets. This study selected TSS > 0.7 and AUC > 0.8 as model selection standards, which are considered to have a high predictive accuracy and low uncertainty (Allouche et al., 2010; Mei et al., 2017). To illustrate the variation in species occurrence probability with the environmental gradient, we plotted the response curve of species with each environmental variable based on the constructed SDMs. We applied a random permutation method to calculate the correlation coefficient between all predictor and estimated variables (Zanardo et al., 2017) to assess the relative importance of each variable in predicting species distributions. Finally, we constructed ensemble models at the species and population levels to predict the potential distributions of the whole species and two populations (EIOS and WPI) under current and future (2050s, 2100s) climate scenarios under RCP 2.6 and RCP 8.5, respectively. Based on the direct outputs from the ensemble models, we produced continuous habitat suitability maps. To better explain habitat suitability, we converted continuous habitat suitability predictions into binary values by maximizing the probability threshold of the TSS (Liu et al., 2013). Here, “prediction existence” signifies the habitat suitability scores that exceed the established threshold, whereas “prediction absence” refers to those scores falling beneath the threshold.

### 2.5 Conservation gap analysis

The global MPA data used in this study was sourced from the World Database on Protected Areas (WDPA, https://www.protectedplanet.net/, accessed on June 15, 2023) (UNEP‐WCMC & IUCN, 2023). First, we conducted an overlay analysis within QGIS 3.30.3 software, including existing MPAs and the distribution range of *T. maxima* at the species level. Subsequently, we conducted a conservation gap analysis to calculate the proportion of existing MPAs covering the range of *T. maxima* and the uncovered spatial area. Based on current and predicted future climate change, this study meticulously assessed the potential habitats of *T. maxima*, aiming to provide a precise basis for future conservation strategies. Particular attention is given to those areas that are currently suitable for the survival of *T. maxima* but may become unsuitable due to climate change in the future. For such areas that are not yet included in existing MPAs, we recommend designating them as Priority Conservation Areas (hereafter “PCAs”) and deploying protection measures in advance to prevent habitat degradation. For areas that are already within MPAs but may become unsuitable in the future, we propose classifying them as Conservation Areas Pending Optimization (hereafter “POCAs”) and recommend adjusting and strengthening protection strategies to ensure the continued survival of *T. maxima* in these regions. These measures will help build a stable and adaptive conservation network, effectively addressing the challenges posed by climate change.

## 3 RESULTS

### 3.1 Niche spatial differences between the two populations

**FIGURE 3:**
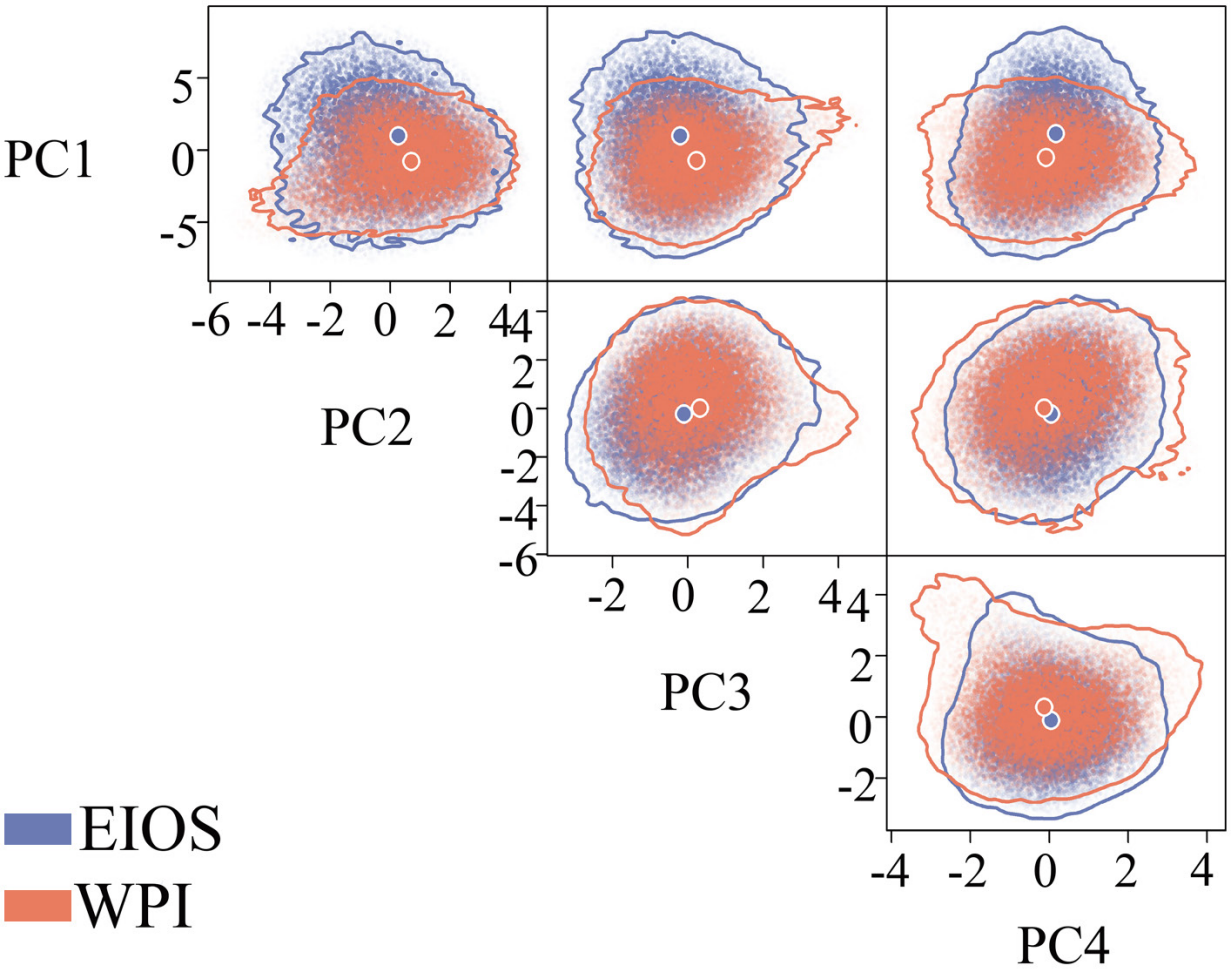
The niches of the two populations of *Tridacna maxima* quantified via four‐dimensional hypervolumes. To visualize the shape and boundary of the hypervolumes in two dimensions, a random selection of 20,000 stochastic points for each hypervolume was used. The large blue and orange points indicate the mean niche position (niche centroid) of EIOS and WPI, respectively.

According to the findings of spatial niche differences between the two populations, the four‐dimensional hypervolume of the EIOS (1992.44) was slightly larger than that of the WPI (1588.38). The niche spatial divergence between the two populations was large (βTotal = 0.61), primarily driven by niche shift (0.28), which accounted for 45.90% of the observed difference, and niche contraction/expansion (0.33), which accounted for 54.10% of the difference (Figure 3, Table S1). Comparison of the positions of the two populations' niche centroids revealed that PCA1 was primarily responsible for the spatial differences in the niches between the EIOS and WPI (Figure 3, Figure S2A), which was primarily caused by mean temperature and salinity (Figure S2B).

### 3.2 Current SDMs projections

**FIGURE 4:**
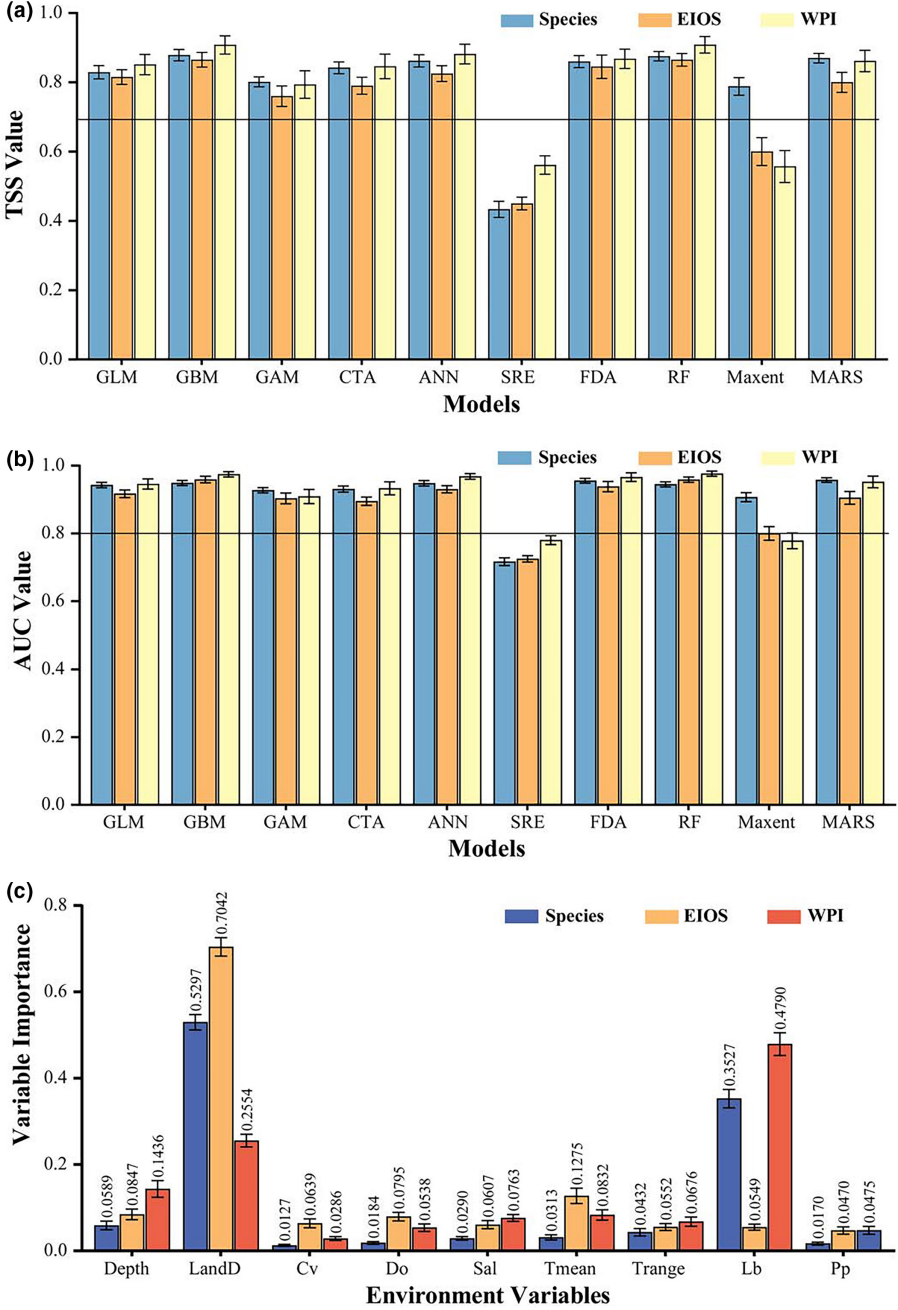
Model evaluation and importance of environmental factors. Predictive abilities of the 10 modeling algorithms in projecting the distribution of *Tridacna maxima* at the population and species levels. (a) The True Skill Statistics (TSS) value; (b) the Area Under the receiver operating characteristic Curve (AUC) value. The black horizontal lines indicate the cutoff values of the AUC (0.8) and TSS (0.7) of the single model used to build the ensemble model. (c) Relative importance of the nine predictor variables in the three ensemble models built at population and species levels. Data are expressed as mean ± standard error. Cv, current velocity; Depth, ocean depth; Do, dissolved oxygen; DTS, distance to shore; Lb, light level at bottom; Pp, phytoplankton; Sal, salinity; Tmean, temperature mean; Trange, temperature mean.

Based on the assessment of model predictive performance using the AUC and TSS values, MaxEnt and SRE were excluded from the ensemble model because their respective AUC and TSS values fell below the established thresholds of 0.8 and 0.7, indicating poor fitting performance. As the AUC and TSS are crucial metrics for evaluating model predictability, failing to meet these standards suggests insufficient predictive capability. As evidenced by relevant research (Araújo & New, 2007; Hao et al., 2020), including high‐performing individual models can significantly enhance the overall predictive power of the ensemble model. Therefore, the exclusion of MaxEnt and SRE was necessary to ensure the reliability and accuracy of the ensemble model. A total of eight modeling algorithms were subsequently selected to construct the weighted ensemble model, with the same high‐performing modeling algorithms retained for building ensemble models at both the population and species levels. The high TSS and AUC values of all three ensemble models indicate their superior predictive performance (Figure 4a,b, Table 2).

**TABLE 2:** Mean values of the true skill statistics (TSS) and the area under the receiver operating characteristic curve (AUC) for the ensemble models built at the species level (species model) and populations level (EIOS model and WPI model).

Ensemble models	TSS	AUC
Species model	0.753	0.892
EIOS model	0.822	0.928
WPI model	0.773	0.886

Abbreviations: EIOS, East Indian Ocean‐South China Sea; WPI, West Pacific‐Indonesia.

The relative importance of each environmental predictor varied among the three ensemble models (Figure 4c). The species‐level model revealed that the distribution of *T. maxima* was primarily influenced by distance to shore and light level at bottom. According to this model, *T. maxima* tended to prefer environments with distance to shore between 0 and 30 km and light level at bottom between 5 and 45 (Figure S3A1,A2). The population‐level model showed that the main factors influencing the potential distribution of *T. maxima* differ between populations. Specifically, the EIOS distribution was most influenced by distance to shore and mean temperature; the occurrence of the EIOS mainly occurred within a distance from land of 40 km and was highest when the mean temperature ranged from 10 to 29°C (Figure S3B1,B2). As for the WPI, light level at bottom and distance to shore were the most significant predictors affecting the distribution of *T. maxima*. This population preferred habitats exhibited light level at bottom above 20 and were located near the coast within approximately 40 km (Figure S3C1,C2).

**FIGURE 5:**
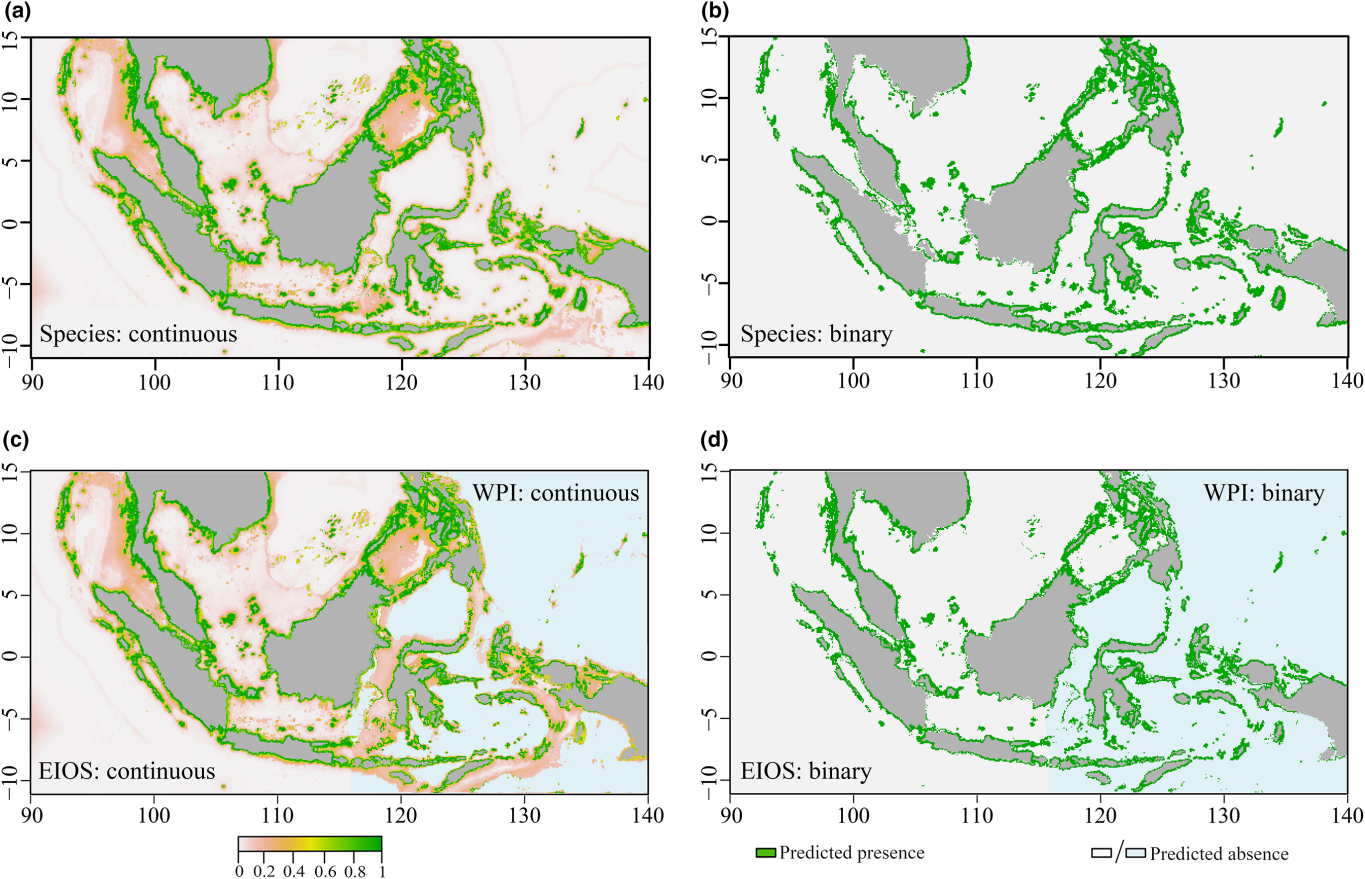
Habitat suitability maps of *Tridacna maxima* predicted by ensemble models under current climate scenarios. Panels (a, b) show the corresponding continuous and binary maps for the species; panels (c, d) show the corresponding maps for EIOS and WPI.

Based on the model results, the distributions and suitable habitats of the two populations of *T. maxima* under current environmental conditions are as follows: EIOS primarily inhabits shallow marine waters near islands such as the Malay Peninsula, Sumatra, Java, Borneo, and the Philippines. On the other hand, WPI is mainly found in the seas surrounding islands such as the Great Sunda Islands, Maluku Islands, and the Philippines (Figure 5c,d).

The results of the SDMs are presented in Figure 5a,b. Both continuous and binary predictions indicate that the suitable habitats predicted by the SDMs are generally similar to the results predicted by the population models, but differences do exist. For example, the predictions of the species model in the marine waters near the Strait of Malacca differ from those of the EIOS model, and the predictions in the waters near Papua New Guinea do not entirely align with those of the WPI model.

### 3.3 Habitat suitability under future climatic scenarios

**FIGURE 6:**
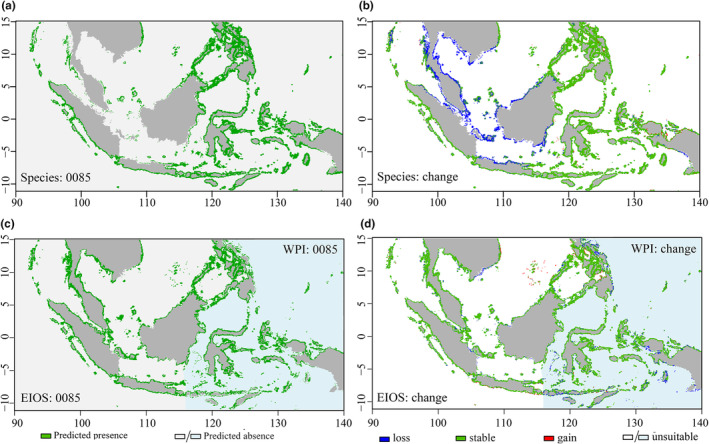
Predicted Habitat Suitability and Changes in *Tridacna maxima* under future climate scenarios. Panels (a, c) are binary maps for each species, and panels (b, d) show the predicted changes in suitable habitats for the 2100s under RCP 8.5 (0085). The category “loss” represents areas projected to be suitable under current climatic conditions but unsuitable under future climatic conditions; “stable” represents areas projected to be suitable under both current and future climatic conditions; “gain” represents areas projected to be unsuitable under current climatic conditions but suitable under future climatic conditions; and “unsuitable” represents areas projected to be unsuitable under current and future climatic conditions.

Large‐scale changes could be caused by climate change scenarios, especially under the pessimistic scenario of uncontrolled greenhouse gas emissions (RCP 8.5), which would lead to significant differences in suitable habitats within the relevant range (Table 3). At the species level, *T. maxima* exhibited a significant loss of suitable habitat in the Malay Peninsula, the southernmost region of the Indochinese Peninsula, the southwestern portion of Borneo, the Strait of Malacca and the majority of the Java Sea (Figure 6a,b). The model projects a 25.86% reduction in the range of *T. maxima* by the end of the 21st century (Table 3).

**TABLE 3:** Size of predicted changes in species range based on population‐level and species‐level models for the middle (2050s) and end (2100s) of the 21st century under RCPs 2.6 and 8.5.

Year RCP	EIOS (%)		WPI (%)		Species (%)	
	2050s	2100s	2050s	2100s	2050s	2100s
RCP 2.6	−2.43	−3.87	−8.60	−8.43	−10.72	−15.13
RCP 8.5	−4.15	−4.17	−10.11	−17.79	−18.49	−25.86

At the population level, both the EIOS and WPI exhibited a decreasing trend in the range of suitable habitats under all climate change scenarios, with the greatest reductions occurring under the RCP 8.5 scenario in the 2100s. For EIOS, the potential habitats of *T. maxima* are mainly stable around the Malay Peninsula, Sumatra, Java, Borneo, and the Philippines, but some habitats are lost along the southeast coast of the Indochinese Peninsula and within the Strait of Malacca, while some suitable habitats are found in the Spratly Islands. For the WPI, the suitable habitats were mainly stable around the Maluku Islands and the surrounding waters of the Sulawesi Sea, while most of the suitable habitats in the eastern waters of the Philippines, Java Sea, and Arafura Sea were lost (Figure 6c,d).

### 3.4 Identifying the *T. maxima* conservation gaps

**FIGURE 7:**
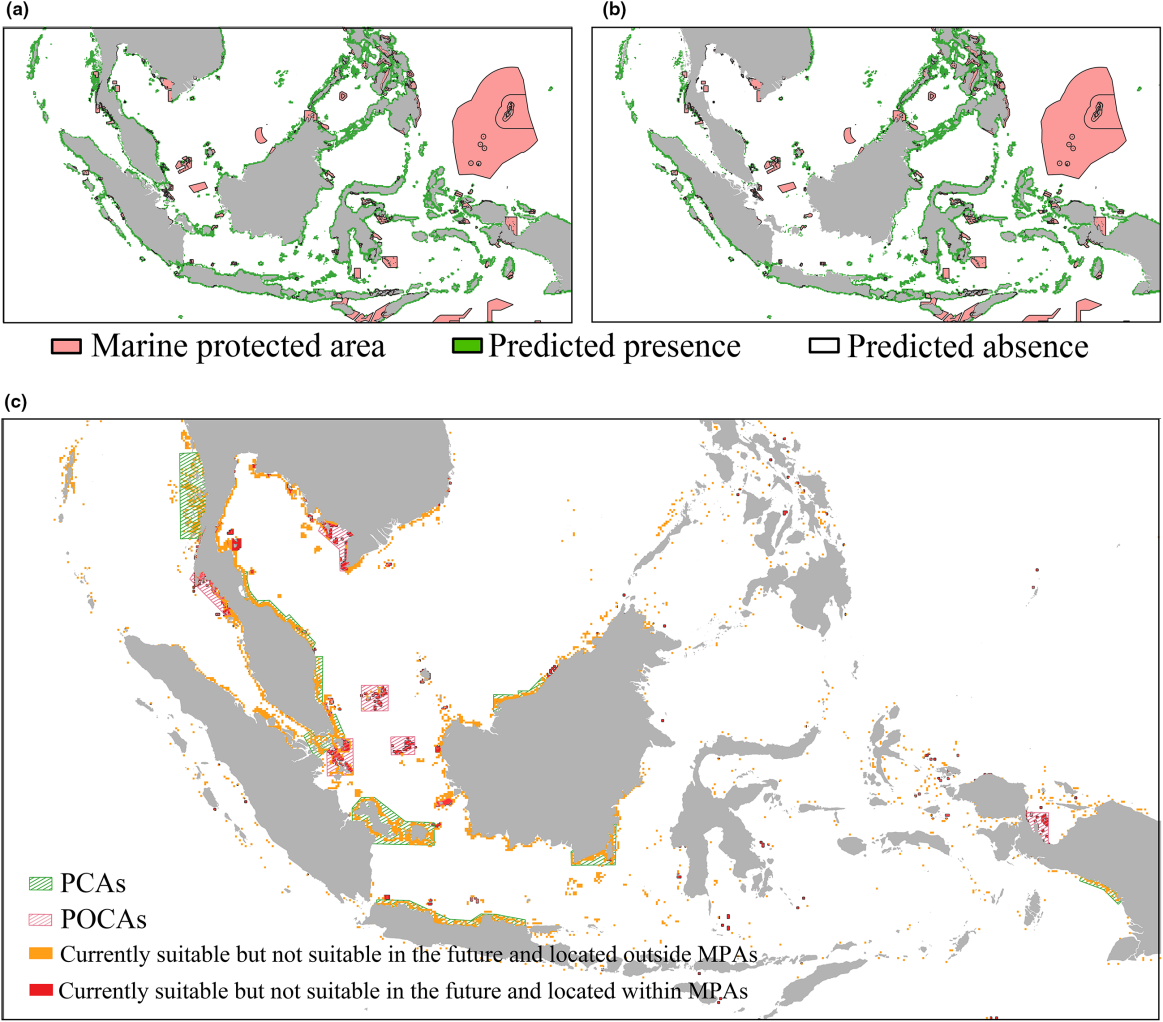
Analysis of marine protected areas (MPAs) Gaps. (a) Analysis of suitable habitats for *Tridacna maxima* in the current climate scenario and existing MPAs gaps. (b) Analysis of suitable habitats for *Tridacna maxima* in the 2100s under the RCP 8.5 climate scenario and existing MPA gaps. (c) Recommendations for Future MPAs. PCAs, Priority Conservation Areas; POCAs, Conservation Areas Pending Optimization; Orange represents: Currently suitable but not suitable in the future and located outside MPAs; Red represents: Currently suitable but not suitable in the future and located within MPAs.

Based on the analysis of conservation gaps, only 244,730.58 km^2^ of the predicted potentially suitable habitats were protected, accounting for 16.10% of the range of *T. maxima*. In other words, approximately 84% of the predicted potentially suitable habitats remain unprotected (Figure 7a). Under future climate scenarios, we overlapped the potential distribution range of *T. maxima* with the existing MPAs. It was found that even with a 15.4% loss in suitable habitat under RCP 8.5 scenario, only 16.18% of the potential distribution range of *T. maxima* would be protected (Figure 7b). Overall, there was little variation in the proportion of conservation gaps under current and future climate scenarios (Table 4). Therefore, urgent efforts are required to undertake habitat conservation initiatives.

**TABLE 4:** *Tridacna maxima* areas and corresponding percentages protected under the current and future climate scenarios.

Climate scenario	Predicted area (km^2^)	Area protected (km^2^)	Percentage of protection
Current	1,519,764.73	244,730.58	16.10
2100s RCP 8.5	1,285,800.49	208,080.73	16.18

In this study, through model predictions analyzing the suitable habitats of *T. maxima* under current and future climate change scenarios, key areas for the establishment or expansion of PCAs were identified. These regions include the southernmost waters near the Bay of Bengal in Myanmar, the eastern coasts of Thailand and West Malaysia, the southern tip of West Malaysia, the waters around the Bangka‐Belitung Province in Indonesia, the northern shores of Java, the surrounding waters of South Kalimantan, the coastal areas along the Arafura Sea in Papua, and the northern coasts of East Malaysia. Although the AquaMaps database indicates that these areas have a high level of species richness, they are currently not adequately protected, and model predictions suggest that these areas may no longer be suitable for the survival of Tridacna maxima in the future. Additionally, for existing protected areas such as the southern Andaman Sea coast of Thailand, the western part of the Mekong Delta in Vietnam, the Riau Islands in Indonesia, and the eastern coast of West Papua Province, which currently provide suitable habitats for *T. maxima*, predicted changes may impact habitat suitability. Therefore, these areas are recommended to be classified as POCAs with adjusted and enhanced protection strategies to ensure the long‐term sustainability of *T. maxima* and its ecosystems (Figure 7c).

## 4 DISCUSSION

### 4.1 SDMs accounting for local adaptation

This study quantified the ecological niches of two populations of *T. maxima* and revealed no significant climate niche conservatism. This finding indicates that the two populations respond differently to environmental gradients and confirms the inapplicability of niche conservatism to this species. Consequently, SDMs were developed for both the EIOS and WPI populations to capture their respective local adaptations. Our research results are consistent with those of previous studies (Li et al., 2022; Sun et al., 2022), as these models once again confirm that the two populations exhibit significantly different responses to climate change predictors. This finding further underscores the importance of considering local adaptation when predicting the potential distribution of species. By thoroughly analyzing the responses of different populations to climate change, we can provide more accurate scientific evidence for conservation and management decisions in the context of climate change.

The species model predicted the potential distribution of *T. maxima* across the entire study area, while the population model produced contrasting results, notably in the EIOS (Figure 6). This suggests that the ecological niche differences between the two populations necessitate the development of separate models. Treating *T. maxima* as a homogeneous unit in the species model obscures local variations in population responses to climatic predictors, thereby compromising the accuracy of the predictions. As emphasized by some studies, species‐level models may underestimate a species' potential habitat when they fail to account for local adaptations (Razgour et al., 2019; Zhang et al., 2021).

In this study, although spatial data models at the species and population levels predicted similar trends of change, the magnitude of range variation differed between the two types of models. Population‐level models provided more encouraging results for the EIOS and WPI, with less loss of suitable habitat. The incorporation of potential local adaptations in the population‐level model has made our climate change estimates less pessimistic. Our findings are consistent with numerous published studies suggesting that adaptive genetic variation within species can reduce their sensitivity to climate change (Collart et al., 2021; Razgour et al., 2019).

### 4.2 Climatic impacts on the *T. maxima*

The two *T. maxima* populations live in places with differing climatic circumstances, which leads to niche space differences and suggests that the populations are affected differently by environmental changes caused by climate change. Our model results indicated that spatial differences in the ecological niches of the EIOS and WPI populations were mainly driven by the expansion/contraction of their ecological niches, with the contributions of the mean water temperature and salinity concentration being the greatest. *T. maxima* is a species of tropical and subtropical water that requires relatively warm temperatures to maintain normal physiological functions and life cycle. Low water temperatures may result in metabolic slowdown, growth retardation, or even mortality. Conversely, high water temperatures may trigger heat stress and other health issues (Brahmi et al., 2021; Dubousquet et al., 2016). Salinity also plays an important role in nutrient absorption, nutrient utilization, and reproductive processes in *T. maxima* (Maboloc & Villanueva, 2017). Furthermore, slight fluctuations in temperature and salinity can instigate intricate alterations in various environmental parameters within aquatic systems. These alterations not only influence nutrient cycling but also significantly impact ecosystem functions, such as energy flow and biodiversity maintenance, as well as the composition of biological communities structure (Viitasalo & Bonsdorff, 2022). The *T. maxima* population may adapt to different climate conditions based on regional environmental factors, leading to population differentiation. Changes in these climate‐induced factors cause the species to alter its current distribution pattern to track its ecological niche. In summary, salinity and temperature, which are pivotal climatic factors, have had significant impacts on the physiological mechanisms, behavioral patterns, and accessibility of food resources in *T. maxima*. These effects have directly or indirectly led to differences in niche space exhibited by various populations in adapting to diverse environments.

The impacts of various climate changes on the potential habitats of species exhibit diverse patterns. According to our species‐level SDM predictions, the Nansha Islands and the Philippines regions possess high ecological adaptability for the EIOS population under the context of climate change, thus holding the potential to become new habitats for them. Furthermore, it is anticipated that most of the existing habitats in these two regions will continue to maintain their suitability. However, this prediction does not indicate that the EIOS population will immediately migrate to these regions, but rather signifies that the climatic conditions in these areas are relatively ideal for the EIOS population. In contrast, the WPI population is expected to remain stable in its current habitats, but the suitable habitats near the Maluku Islands and the Philippine Sea may decrease due to climate change, suggesting to some extent that the WPI population may face greater challenges in adapting to climate change. Overall, climate change‐induced increases in water temperature and changes in salinity have further exacerbated population niche differences in *T. maxima*. The underlying reason for this divergence is the different responses of different populations to climate change.

### 4.3 Accuracy of model prediction

Integrated habitat suitability models constructed using a weighted integration approach can improve model prediction accuracy and preserve interpretability, particularly for rare species (Breiner et al., 2015). The high AUC and TSS values (AUC > 0.85, TSS > 0.94) across all three models indicate that the predictions were very accurate representations of the *T. maxima* distributions in the present and the future under different climate scenarios. According to earlier research, giant clams, such as *T. maxima*, are a flagship taxon for efforts to conserve coral reefs because they support overall reef biodiversity and functionality (Killam et al., 2023; Lee et al., 2022). This consistency can be attributed to the interaction and dependency between *T. maxima* and coral reefs.

Although the results align with expectations, the SDMs approach for predicting the range of *T. maxima* still has certain drawbacks. In our study, the occurrence data for *T. maxima* primarily rely on online datasets. To improve or validate the predictive capabilities of SDMs, it is necessary to collect geographically or temporally independent data (Bahn & McGill, 2013). Given the rarity and resource limitations of *T. maxima*, emerging environmental DNA technology can be applied to determine the presence of *T. maxima* within potential distribution areas. These data will provide valuable information for future habitat distribution model research. Furthermore, existing research evidence suggests that *T. maxima* exhibits two evolutionary branches within the spatial scale of our study region (Nuryanto & Kochzius, 2009). However, more detailed taxonomic classifications may provide more accurate predictions. Therefore, future studies can further explore the validity of this hypothesis by conducting additional genetic research on *T. maxima*. Moreover, this study utilized projected data from AOGCMs as future environmental data upon which our research is based. It is important to note that these data are not directly derived from objective observations but rather represent inferred outcomes of future environmental conditions based on model simulations. In this study, we acknowledged that assuming that *T. maxima* had infinite dispersal capabilities may have overestimated its potential distribution range. However, dispersal ability is an important factor in determining a species' potential distribution. In reality, due to a range of barriers, such as water flow and temperature gradients, *T. maxima* may not be able to disperse to all suitable areas (Hui et al., 2016). Therefore, future prediction models should consider these factors to more accurately describe the actual distribution of the species.

### 4.4 *T. maxima* conservation and adaptative management

*Tridacna maxima*, commonly known as the giant clam, is a key species in marine ecosystems that plays significant roles in various ecological processes and occupies an important position in the seabed food web (Rong et al., 2021). Its benthic lifestyle and filter‐feeding habits have a marked influence on the water quality and sediment characteristics of the surrounding environment, which may alter the structure and function of benthic communities such as zooxanthellae (Hoeksema, 2007). Furthermore, the presence of giant clams provides habitats and food sources for other marine organisms, thus playing an essential role in supporting the stability and functionality of the entire ecosystem. The protection of suitable habitats for giant clams not only contributes to maintaining the stability and survival status of the species, but also indirectly safeguards the stability of the surrounding biological communities and ecosystems (Bouma et al., 2009). Therefore, in the long run, determining how climate change will affect the habitat suitability of *T. maxima* and developing conservation and management plans to address these changes are highly valuable for the species and its related ecosystems.

Marine protected areas have been proven to be the most effective tools for protecting endangered species and maintaining ecosystem services (Grorud‐Colvert et al., 2021). Although they are effective, our analysis of the spatial overlap between existing MPAs and the potential distribution of *T. maxima* indicates that only 16.10% of the area is currently protected, leaving approximately 84% unprotected. This finding suggests that the established network of protected areas still falls short in terms of the area coverage of the maximum potential habitat for *T. maxima*. Therefore, this study underscores the importance of establishing or expanding PCAs and POCAs to protect *T. maxima* and its critical habitats. Our research, combined with data from the AquaMaps database (Shao et al., 2014), suggests that the proposed priority conservation areas are rich in biodiversity yet under protection, highlighting the significance of establishing protected areas for marine ecological conservation. As a key species in coral reef ecosystems, *T. maxima* plays an irreplaceable role in maintaining ecosystem health and functionality (Guibert et al., 2020; Waters et al., 2013). By prioritizing the protection of these high‐biodiversity yet insufficiently protected areas, we can effectively mitigate the negative impacts of climate change on these habitats and enhance the resilience and stability of the entire marine ecosystem. Furthermore, the optimization of existing protected areas should be based on detailed scientific research and ecological assessments.

POCAs each possess unique ecosystems and biodiversity. For instance, the coastal regions along the Andaman Sea are rich in coral reef and seagrass bed ecosystems, which serve as crucial habitats for numerous marine species (Raghunathan & Venkataraman, 2015; Raghuraman et al., 2013); the western coastal areas of the Mekong Delta are vital wetland ecosystems that play a key ecological role for waterbirds, fish, and other aquatic organisms (Campbell, 2012). However, as environmental conditions rapidly change, the conservation strategies for these areas may need to be adjusted. By employing spatial analysis and ecological modeling tools, the ecological sensitivity and conservation value of these areas can be precisely assessed, allowing for the redefinition of protected area boundaries and the adjustment of management measures (Noble et al., 2020). This science‐based approach to conservation planning and management adjustments not only ensures the protection of key species such as *T. maxima* but also guarantees the continued provision of ecosystem services, achieving long‐term biodiversity conservation and stable ecosystem functions.

Finally, it must be emphasized that while establishing MPAs and implementing other conservation measures are crucial for protecting the current and future suitable habitats of *T. maxima* from human activities, the challenges these efforts face are increasing with the intensification of global climate change. The latest IPCC report (IPCC, 2023) revealed a gradual reduction in the optimal habitats for *T. maxima*, indicating a potential risk of extinction for the species in the future. Therefore, in addition to existing conservation measures, reducing anthropogenic greenhouse gas emissions and strengthening climate change mitigation measures through international cooperation are particularly important. These steps are fundamental to ensuring the sustainable development of these species and marine ecosystems. Achieving this goal requires the cooperation of global governments, environmental organizations, and scientific institutions to implement stringent emission restrictions and extensive ecological conservation measures (Fawzy et al., 2020), addressing the ecological challenges posed by climate change and thereby maintaining the health and prosperity of global biodiversity.

## 5 CONCLUSION

In summary, this study represents the first attempt to evaluate the potential impact of climate on the distribution of *T. maxima* in the Indo‐Pacific core region, while taking into account local adaptability. The differential responses of the two *T. maxima* populations to environmental predictor variables indicate that population‐level SDMs are more reliable than species‐level SDMs. Moreover, to better protect *T. maxima*, further efforts to expand the conservation of its habitat and expedite the establishment or expansion of existing MPAs are recommended. In future planning of protected areas, priority should be given to covering the critical habitats of *T. maxima*, particularly the currently unprotected areas. Furthermore, with reference to international recommendations for achieving the target of marine biodiversity conservation coverage by 2030, there is a pressing need to significantly enhance the spatial coverage of protected areas for *T. maxima* habitats. This study provides current and future predictive habitat suitability maps, which can sever as robust tools for identifying regions with the highest conservation potential. Future research should integrate other analytical methods and diverse data sources to improve the predictive capability of *T. maxima*'s potential distribution, thereby providing more accurate information for relevant conservation and management efforts.

## AUTHOR CONTRIBUTIONS

**Shenghao Liu:** Conceptualization (lead); formal analysis (equal); funding acquisition (lead); writing – original draft (lead). **Tingting Li:** Data curation (lead); formal analysis (lead); methodology (lead); visualization (lead). **Bailin Cong:** Data curation (equal); formal analysis (equal); methodology (equal). **Leyu Yang:** Data curation (equal); formal analysis (equal). **Zhaohui Zhang:** Conceptualization (equal); project administration (lead); supervision (lead). **Linlin Zhao:** Conceptualization (equal); funding acquisition (equal); writing – review and editing (lead).

## CONFLICT OF INTEREST STATEMENT

The authors declare that they have no conflicts of interest regarding this research.

## Supporting information


Data S1

Figure S1

## Data Availability

The raw data used in this study are available through the following links: the Global Biodiversity Information Facility (GBIF, https://www.gbif.org/), iNaturalist (https://www.inaturalist.org/), and the Ocean Biogeographic Information System (OBIS, https://obis.org/). Bio‐ORACLE (https://bio‐oracle.org/downloads‐to‐email.php) and Global Marine Environment Datasets (GMED, https://gmed.auckland.ac.nz/download.html). The analyzed code can be found in the RCode file.
