# Supplementary material for: Unveiling the suitable habitats and future conservation strategies of Tridacna maxima in the Indo‐Pacific core area based on species distribution model
Source: Ecol Evol. 2024 Sep 4;14(9):e70187. doi: 10.1002/ece3.70187 (PMC11372821; doi:10.1002/ece3.70187)
Supplement: Supplementary file 1 — Data S1 [file ECE3-14-e70187-s001.pdf]

```
##一 preparation

  #create a new directory
  dir.create("D:/SDM_Tridacna/Standardization/setp1_data_download")
  #Set the current workspace
  setwd("D:/SDM_Tridacna/Standardization/setp1_data_download")
  #View the current workspace
  getwd()

#Batch Download Package
packages=c("ade4","factoextra","magrittr","cowplot","ggplot2",
           "dplyr","tidyr","maps","leaflet")
ipak <- function(pkg){
  new.pkg <- pkg[!(pkg %in% installed.packages()[, "Package"])]
  if (length(new.pkg))
    install.packages(new.pkg, dependencies = TRUE)
  sapply(pkg, require, character.only = TRUE)    #批量下载包
}
ipak(packages)
#Package loading
{
  library(sp)
  library(rgdal)
  library(dismo)
  library(readxl)
  library(sdmpredictors)
  library(maptools)
  library(raster)
  library(biomod2)
  library(ade4)
  library(factoextra)
  library(magrittr)
  library(cowplot)
  library(ggplot2)
  library(dplyr)
  library(tidyr)
  library(maps)
  library(leaflet)
  library(ecospat)
  library(car)
  library(caret)
  library(ggcorrplot)
  library(corrplot)
  library(plyr)
```

```
  library(Rmisc)
  library(gridExtra)
  library(ggalt)
  library(xlsx)
}
####step1 data preocessing
{
colu=read.csv("summary_yuanshi.csv")
colnames(colu) <-c("spec", "lon", "lat")
summary_yuanshi <- colu
##To repeat
dups2 <- duplicated(summary_yuanshi[, c('spec','lon', 'lat')])
sum(dups2)
summary_dup <- summary_yuanshi[!dups2, ]
##Remove missing values
summary_dup[-which(is.na(summary_dup$lon)),]
summary_dup_drop=drop_na(summary_dup,lon)
write.csv(summary_dup_drop, file = "summary.csv")

#spThin
colu=read.csv("summary.csv")

library(spThin)
thinned_dataset_full <-
  thin( loc.data =colu,
        lat.col = "lat", long.col = "lon",
        spec.col = "spec",
        thin.par = 9.2, reps = 1,
        locs.thinned.list.return = TRUE,
        write.files = TRUE,
        max.files = 1,
        out.dir  =  "D:/SDM_Tridacna/Standardization/TA",  out.base  =
"summary",
        write.log.file = TRUE,
        log.file = "summary.csv" )
thinned_dataset_full<-thinned_dataset_full[[1]]
thinned_dataset_full<-thinned_dataset_full[1:1460,]
A51_cl=thinned_dataset_full[,1:2]
names(A51_cl)<-c('lon', 'lat')
write.csv(A51_cl, file = "A51_cl.csv")

##Building projection----
data("wrld_simpl")  #World Map Reference System
coordinates(A51_cl) <- c(1,2)
```

```
plot(A51_cl)
crs(A51_cl) <- crs(wrld_simpl)
plot(wrld_simpl)
##Find the ones in the ocean----
ovr <- over(A51_cl, wrld_simpl)
cntr <- ovr$NAME
i <- which(is.na(cntr))
A51=A51_cl[i,]
A51=as.data.frame(A51)

}
##draw designs
mapworld<-borders("world",colour = "black",fill ="grey70",lty=3,lwd
=0.42)
p<-ggplot(A51, aes(x = lon, y = lat))+mapworld+ylim(-50,50) +xlim(-
180,180)+
  theme_bw()+
  geom_point(colour ="red",size =0.5)+
  scale_y_continuous(expand = c(0.05,0))+
  scale_x_continuous(expand = c(0.02,0))+
  theme(panel.grid.major = element_blank(),panel.grid.minor =
element_blank(),
        panel.border =
element_rect(fill=NA,color="black",size=0.8,linetype="solid"),
        axis.title.x = element_text(size=14), axis.title.y =
element_text(size=14),
        axis.text.x = element_text(size=10), axis.text.y =
element_text(size=10))+
  coord_fixed()
p
ggplotly(p)

###二、Import environment variables----
{
  ##present---
  depthbm=readGDAL("environment/present/Depth2.tif")
  landDbm=readGDAL("environment/present/Land_Distance2.tif")
  cvbm=readGDAL("environment/present/Current_Velocity.tif")
  dobm=readGDAL("environment/present/Dissolved_oxygen.tif")
  salbm=readGDAL("environment/present/Salinity.tif")
  tmeanbm=readGDAL("environment/Present/Temperature_Mean.tif")
  trangebm=readGDAL("environment/Present/Temperature_Range.tif")
  lbbm=readGDAL("environment/Present/Light_bottom.tif")
  Ppbm=readGDAL("environment/present/Primary_productivity.tif")
```

```
#raster
depthbm=raster(depthbm)
landDbm=raster(landDbm)
cvbm=raster(cvbm)
dobm=raster(dobm)
salbm=raster(salbm)
tmeanbm=raster(tmeanbm)
trangebm=raster(trangebm)
lbbm=raster(lbbm)
Ppbm=raster(Ppbm)

##Define a scope
e<-extent(90 , 140 , -11, 15 )
s<-raster(e, nrows=312, ncols=600)
#crop by e
depthbm=crop(depthbm,e)
landDbm=crop(landDbm,e)
cvbm=crop(cvbm,e)
dobm=crop(dobm,e)
salbm=crop(salbm,e)
tmeanbm=crop(tmeanbm,e)
trangebm=crop(trangebm,e)
lbbm=crop(lbbm,e)
Ppbm=crop(Ppbm,e)

##Transfer values between mismatched grid objects (based on origin and resolution)
depthbm=resample(depthbm,s,method="ngb")
landDbm=resample(landDbm,s,method="ngb")
cvbm=resample(cvbm,s,method="ngb")
dobm=resample(dobm,s,method="ngb")
salbm=resample(salbm,s,method="ngb")
tmeanbm=resample(tmeanbm,s,method="ngb")
trangebm=resample(trangebm,s,method="ngb")
lbbm=resample(lbbm,s,method="ngb")
Ppbm=resample(Ppbm,s,method="ngb")

#show
evir_now=stack(depthbm,landDbm,cvbm,dobm,salbm,tmeanbm,trangebm,lbbm,Ppbm)
plot(evir_now)
```

```
names(evir_now)=c("depthbm","landDbm","cvbm","dobm","salbm","tmeanbm",
"trangebm","lbbm","Ppbm")
  plot(evir_now)
}

## 三、Pearson correlation and VIF analysis of environmental factors--
--
{
  # Extract environment variables for sampling points
  env_jh                                                              <-
list(depthbm,landDbm,cvbm,dobm,salbm,tmeanbm,trangebm,lbbm,Ppbm)
  env_name<-
c("depthbm","landDbm","cvbm","dobm","salbm","tmeanbm","trangebm","lbbm
","Ppbm")
  env_extract <- list()
  for (i in 1:length(env_name)) {
    env_extract[[i]]  <-  raster::extract(raster::stack(env_jh[[i]]),
A51[2:3])
  }
  env_extract <- data.frame(na.omit(data.frame(env_extract)))
  names(env_extract) <- env_name

  present  <-  as.matrix(seq(from  =  1.0,  to  =  1.0,  length.out  =
nrow(env_extract)))
  env_extract1 <- cbind(present, env_extract)
  # Analysis+Drawing
  # pearson correlation analysis
  env.cor <- round(cor(env_extract, method = "pearson"), 3)
  library(ggcorrplot)
  env.p <- round(cor_pmat(env_extract, method = "pearson"), 3)
  library(corrplot)
  par(mfrow = c(1, 1), mar = c(2, 2, 1, 1))
  cor.plot <- corrplot(corr = env.cor, type = "upper", tl.pos = "tp",
tl.col = "black", tl.cex = 1.2, cl.cex = 1, p.mat = env.p, insig =
"label_sig", sig.level = c(.01, .05), pch.cex = 1.5, pch.col = "black",
order = "original")
  cor.plot <- corrplot(
    corr = env.cor, type = "lower", add = TRUE, method = "number",
    tl.pos = "n", tl.col = "black",
    col = "black", tl.cex = 1.3, diag = FALSE, cl.pos = "n", pch.col =
"black", # cl.cex图例字体大小
    number.cex = 1, number.font = 1, order = "original"
  )
 #Scatter/collinearity
```

```
  library(car)
  scatterplotMatrix(env_extract, main = "correlation")

  # VIF
  result <- lm(present ~ ., data = env_extract1)
  env_extract1
  myStep <- step(result, direction = "both")
  summary(myStep)
  vif(myStep)
}


getwd()

library(hypervolume)
library("corrplot")
library("FactoMineR")
library("factoextra")
library(sp)
library(rgdal)
library(dismo)
library(readxl)
library(sdmpredictors)
library(maptools)
library(raster)
library(ade4)
library(factoextra)
library(magrittr)
library(cowplot)
library(ggplot2)
library(dplyr)
library(tidyr)
library(maps)
library(leaflet)
library(BAT)

colu=read.csv("EIOS202.csv")

library(spThin)
thinned_dataset_full <-
  thin( loc.data =colu,
        lat.col = "lat", long.col = "lon",
        spec.col = "spec",
        thin.par = 9.2, reps = 1,
```

```
        locs.thinned.list.return = TRUE,
        write.files = TRUE,
        max.files = 1,
        out.dir                                                    =
"D:/SDM_Tridacna/Standardization/setp3_Subregion_Niche",   out.base   =
"EIOS202_thinned_data",
        write.log.file = TRUE,
        log.file = "EIOS202_thinned_data.CSV" )

colu2=read.csv("WPI684.csv")

library(spThin)
thinned_dataset_full <-
  thin( loc.data =colu2,
        lat.col = "lat", long.col = "lon",
        spec.col = "spec",
        thin.par = 9.2, reps = 1,
        locs.thinned.list.return = TRUE,
        write.files = TRUE,
        max.files = 1,
        out.dir                                                    =
"D:/SDM_Tridacna/Standardization/setp3_Subregion_Niche",   out.base   =
"WPI684_thinned_data",
        write.log.file = TRUE,
        log.file = "WPI684_thinned_data.CSV" )

depthbm=readGDAL("environment/present/Depth2.tif")
landDbm=readGDAL("environment/present/Land_Distance2.tif")
cvbm=readGDAL("environment/present/Current_Velocity.tif")
dobm=readGDAL("environment/present/Dissolved_oxygen.tif")
salbm=readGDAL("environment/present/Salinity.tif")
tmeanbm=readGDAL("environment/Present/Temperature_Mean.tif")
trangebm=readGDAL("environment/Present/Temperature_Range.tif")
lbbm=readGDAL("environment/Present/Light_bottom.tif")
Ppbm=readGDAL("environment/present/Primary_productivity.tif")


depthbm=raster(depthbm)
landDbm=raster(landDbm)
cvbm=raster(cvbm)
dobm=raster(dobm)
salbm=raster(salbm)
tmeanbm=raster(tmeanbm)
trangebm=raster(trangebm)
```

```
lbbm=raster(lbbm)
Ppbm=raster(Ppbm)

e<-extent(90 , 140 ,-11,15 )
s<-raster(e, nrows=312, ncols=600)

depthbm=crop(depthbm,e)
landDbm=crop(landDbm,e)
cvbm=crop(cvbm,e)
salbm=crop(salbm,e)
dobm=crop(dobm,e)
tmeanbm=crop(tmeanbm,e)
trangebm=crop(trangebm,e)
lbbm=crop(lbbm,e)
Ppbm=crop(Ppbm,e)


depthbm=resample(depthbm,s,method="ngb")
landDbm=resample(landDbm,s,method="ngb")
cvbm=resample(cvbm,s,method="ngb")
salbm=resample(salbm,s,method="ngb")
dobm=resample(dobm,s,method="ngb")
tmeanbm=resample(tmeanbm,s,method="ngb")
trangebm=resample(trangebm,s,method="ngb")
lbbm=resample(lbbm,s,method="ngb")
Ppbm=resample(Ppbm,s,method="ngb")


evir_now=stack(depthbm,landDbm,cvbm,salbm,dobm,tmeanbm,trangebm,lbbm,P
pbm)
names(evir_now)=c("depthbm","landDbm","cvbm","salbm","dobm","tmeanbm",
"trangebm","lbbm","Ppbm")
plot(evir_now)

##Import Data----
colu1=read.csv("D:/SDM_Tridacna/Standardization/setp3_Subregion_Niche/
EIOS202_thinned_data_thin1.csv")
A51_cl=colu1[,2:3]
##To repeat---
dups2 <- duplicated(A51_cl[, c('lon', 'lat')])
sum(dups2)
A51_cl <- A51_cl[!dups2, ]
##Remove missing values----
A51_cl[-which(is.na(A51_cl$lon)),]
```

```
A51_cl=drop_na(A51_cl,lon)
##Building projection----
data("wrld_simpl")
coordinates(A51_cl) <- c(1,2)
crs(A51_cl) <- crs(wrld_simpl)
##Find the ones in the ocean----
ovr <- over(A51_cl, wrld_simpl)
cntr <- ovr$NAME
i <- which(is.na(cntr))
A51=A51_cl[i,]
A51=data.frame(A51)
gg1 <-  raster::extract(evir_now,A51)
gg1 <- na.omit(gg1)
name<-c("EIOS")
name <- as.data.frame(name)
gg1 <- cbind(gg1,name)
#gg1[,8] <- as.factor(gg1[,8])
gg1[,10] <- as.factor(gg1[,10])
gg1

colu2=read.csv("D:/SDM_Tridacna/Standardization/setp3_Subregion_spThin
_1/WPI684_thinned_data_thin1.csv")
A52_cl=colu2[,2:3]
##To repeat----
dups3 <- duplicated(A52_cl[, c('lon', 'lat')])
sum(dups3)
A52_cl <- A52_cl[!dups3, ]
##Remove missing values----
A52_cl[-which(is.na(A52_cl$lon)),]
A52_cl=drop_na(A52_cl,lon)
##Building projection----
data("wrld_simpl")
coordinates(A52_cl) <- c(1,2)
crs(A52_cl) <- crs(wrld_simpl)
##Find the ones in the ocean----
ovr <- over(A52_cl, wrld_simpl)
cntr <- ovr$NAME
i <- which(is.na(cntr))
A52=A52_cl[i,]
A52=data.frame(A52)
gg2 <-  raster::extract(evir_now,A52)
gg2 <- na.omit(gg2)
name<-c("WPI")
name <- as.data.frame(name)
```

```
gg2 <- cbind(gg2,name)
#gg2[,8] <- as.factor(gg2[,8])
gg2[,10] <- as.factor(gg2[,10])
gg2
gg <- rbind(gg1,gg2)


# PCA
res.pca <- PCA(gg[,c(1:9)], graph = FALSE)
res.pca
var<- get_pca_var(res.pca)
var
var$coord
ind <- get_pca_ind(res.pca)
ind
ind$coord
#Visualize the relationship between variables and principal components
head(var$cos2)
#  is.corr  indicates  that  the  input  matrix  is  not  a  correlation
coefficient matrix
corrplot(var$cos2, is.corr=FALSE)
#Graph the contribution of variables to principal components
head(var$contrib, 4)
corrplot(var$contrib, is.corr=FALSE)
#The contribution of each variable to the first principal component
fviz_contrib(res.pca, choice = "var", axes = 1, top = 10)
#The contribution of each variable to the second principal component
fviz_contrib(res.pca, choice = "var", axes = 2, top = 10)
fviz_contrib(res.pca, choice = "var", axes = 3, top = 10)
fviz_contrib(res.pca, choice = "var", axes = 4, top = 10)
write.csv(res.pca$var$contrib,file = "Composition of PCA axis.csv")
#Percentage of Interpretation Degree of Principal Component Axis
eig.val <- get_eigenvalue(res.pca)
eig.val
write.csv(eig.val,file = "PCA interpretability.csv")

fviz_screeplot(res.pca,geom = c("bar","line"),ncp=10,addlabels = TRUE)
ggg <- ind$coord

a <- gg[,10]
a <- as.data.frame(a)
ggg <- cbind(a,ggg)
ggg <- ggg[,-6]
ggg
```

```
range(ggg$Dim.1)
range(ggg$Dim.2)
range(ggg$Dim.3)
range(ggg$Dim.4)

ggg <- subset(ggg,ggg$Dim.1<5)
ggg <- subset(ggg,ggg$Dim.1>-5)
ggg <- subset(ggg,ggg$Dim.2<4.5)
ggg <- subset(ggg,ggg$Dim.3<4)
ggg <- subset(ggg,ggg$Dim.3>-3.6)
ggg <- subset(ggg,ggg$Dim.4<3.2)

species_list = as.character(unique(ggg$a))
num_species = length(species_list)
trait_axes <-  c("Dim.1","Dim.2","Dim.3","Dim.4")

# compute hypervolumes for each species
hv_finches_list = new("HypervolumeList")
hv_finches_list@HVList = vector(mode="list",length=num_species)
for (i in 1:num_species)
{
  # keep the trait data
  data_this_species = ggg[ggg$a==species_list[i],trait_axes]
  # log-transform to rescale
  #data_this_species_log <- log10(data_this_species)

  # make a hypervolume using auto-bandwidth
  hv_finches_list@HVList[[i]]                                        <-
hypervolume_gaussian(data_this_species,

name=as.character(species_list[i]),
                                                    kde.bandwidth   =
2*estimate_bandwidth(data_this_species),
                                                    verbose=FALSE)
}

# compute all pairwise overlaps
overlap = matrix(NA, nrow=num_species, ncol=num_species)
dimnames(overlap)=list(species_list, species_list)
for (i in 1:num_species)
{
  for (j in i:num_species)
  {
    if (i!=j)
```

```
    {
      # compute set operations on each pair
      this_set      =      hypervolume_set(hv_finches_list@HVList[[i]],
hv_finches_list@HVList[[j]], check.memory=FALSE)
      # calculate a Sorensen overlap index (2 x shared volume / sum of
|hv1| + |hv2|)
      overlap[i,j]                                                      =
hypervolume_overlap_statistics(this_set)["sorensen"]
    }
  }
}
tiff(file = "N-dimensional Hypervolume.tiff",width = 5000, height =
3829,res = 600,compression ="jpeg")
plot(hv_finches_list,quantile.requested=0.5,show.contour=T,
     show.data=F, num.points.max.random = 20000,num.points.max.data =
2000,

contour.type='kde',contour.kde.level=0.005,colors=c("#6F80BE","#F47E62
"),names=c("PC1","PC2","PC3", "PC4"),
     point.alpha.min=0,contour.lw=2,cex.centroid=2,
     show.legend=F,cex.legend=3,cex.axis=1,cex.names=2,reshuffle   =F)
legend("bottomleft",legend       =       c("EIOS","WPI"),fill       =
c("#6F80BE","#F47E62"),bty="n",
       cex = 1.5)
dev.off()


hv1_thinned1      =      hypervolume_thin(hv_finches_list@HVList[[1]],
num.points=1000)
plot(hv1_thinned1)
hv1_thinned2      =      hypervolume_thin(hv_finches_list@HVList[[2]],
num.points=1000)
plot(hv1_thinned2)
plot(hypervolume_join(hv1_thinned1,
hv1_thinned2),quantile.requested=0.5,
     col=c('red','green'), names=c("PC1","PC2","PC3", "PC4"),
     num.points.max.random = 10000,
     show.legend=TRUE,cex.names=1.5,contour.lwd=2)

# show all hypervolumes

tiff(file = "N-dimensional Hypervolume.tiff",width = 5000, height =
3829,res = 600,compression ="jpeg")
plot(hv_finches_list,quantile.requested=0.5,show.contour=T,
```

```
      show.data=F,  num.points.max.random = 20000,num.points.max.data = 1000,

contour.type='kde',colors=c("#6F80BE","#F47E62"),names=c("PC1","PC2","PC3", "PC4"),
      point.alpha.min=0,contour.lw=2,cex.centroid=2,
      show.legend=F,cex.legend=3,cex.axis=1,cex.names=2,reshuffle   =F)
legend("bottomleft",legend       =       c("EIOS","WPI"),fill       = c("#6F80BE","#F47E62"),bty="n",
        cex = 1.5)
dev.off()

kernel.alpha(hv_finches_list)
kernel.beta(hv_finches_list)
hv_set         <-         hypervolume_set(hv_finches_list@HVList[[1]], hv_finches_list@HVList[[2]], check.memory=FALSE)
hypervolume_overlap_statistics(hv_set)
kernel.similarity(hv_finches_list)

###四prediction:TS-future
#(一)Package loading
{
  library(sp)
  library(rgdal)
  library(raster)
  library(dismo)
  library(readxl)
  library(sdmpredictors)
  library(maptools)
  library(biomod2)
  library(ade4)
  library(ggplot2)
  library(factoextra)
  library(magrittr)
  library(cowplot)
  library(dplyr)
  library(tidyr)
  library(maps)
  library(leaflet)
  library(ecospat)
  library(car)
  library(lattice)
  library(caret)
  library(ggcorrplot)
```

```
  library(corrplot)
  library(plyr)
  library(Rmisc)
  library(gridExtra)
  library(ggalt)
  library(xlsx)
}
## (二 ) Import distribution data----

#Evolutionary Branch 1+2
colu=read.csv("EIOS_WPI_thinned_data_thin1.csv")
A51_cl=colu[,2:3]
##To repeat---
dups2 <- duplicated(A51_cl[, c('lon', 'lat')])
sum(dups2)
A51_cl <- A51_cl[!dups2, ]
##Remove missing values ----
A51_cl[-which(is.na(A51_cl$lon)),]
A51_cl=drop_na(A51_cl,lon)
##Building projection----
data("wrld_simpl")
coordinates(A51_cl) <- c(1,2)
crs(A51_cl) <- crs(wrld_simpl)
##Find the ones in the ocean----
ovr <- over(A51_cl, wrld_simpl)
cntr <- ovr$NAME
i <- which(is.na(cntr))
A51=A51_cl[i,]
A51=data.frame(A51)

###draw designs----
par(mfrow=c(1,1),mar=c(2,2,2,2))
tiff(file = "Species distribution points.tiff",width = 5124, height =
3150,res = 600,compression ="jpeg")
map('world2',xlim=c(90, 140), ylim=c(-11,15),col = c("grey70"),fill =
TRUE,border="black",  lwd=0.5, lty=3)
#map('world2',xlim=c(90,        140),        ylim=c(-11,15),col        =
c("lightyellow"),fill = TRUE,border="black",  lwd=1, lty=3)
axis(1); axis(2); box()
points(A51[,1:2], pch=20, col="red",cex=0.35)
#points(A51[,1:2], pch=20, col="#A9192A",cex=0.25)
points(A51[,1:2], pch=20, col="#189118",cex=0.35)
dev.off()
```

```
## (三) Import environment variables----
##present---
depthbm=readGDAL("environment/present/Depth2.tif")
landDbm=readGDAL("environment/present/Land_Distance2.tif")
cvbm=readGDAL("environment/present/Current_Velocity.tif")
salbm=readGDAL("environment/present/Salinity.tif")
tmeanbm=readGDAL("environment/Present/Temperature_Mean.tif")
trangebm=readGDAL("environment/Present/Temperature_Range.tif")
dobm=readGDAL("environment/present/Dissolved_oxygen.tif")
lbbm=readGDAL("environment/Present/Light_bottom.tif")
Ppbm=readGDAL("environment/present/Primary_productivity.tif")

#raster
depthbm=raster(depthbm)
landDbm=raster(landDbm)
cvbm=raster(cvbm)
salbm=raster(salbm)
tmeanbm=raster(tmeanbm)
trangebm=raster(trangebm)
dobm=raster(dobm)
lbbm=raster(lbbm)
Ppbm=raster(Ppbm)

##Define a scope
e<-extent(90 , 140 , -11, 15 )
s<-raster(e, nrows=312, ncols=600)
#crop by e
depthbm=crop(depthbm,e)
landDbm=crop(landDbm,e)
cvbm=crop(cvbm,e)
salbm=crop(salbm,e)
tmeanbm=crop(tmeanbm,e)
trangebm=crop(trangebm,e)
dobm=crop(dobm,e)
lbbm=crop(lbbm,e)
Ppbm=crop(Ppbm,e)

#env=reseample(env,s,method="ngb)
#Transfer values between mismatched grid objects (based on origin and
resolution)
depthbm=resample(depthbm,s,method="ngb")
landDbm=resample(landDbm,s,method="ngb")
```

```
cvbm=resample(cvbm,s,method="ngb")
salbm=resample(salbm,s,method="ngb")
tmeanbm=resample(tmeanbm,s,method="ngb")
trangebm=resample(trangebm,s,method="ngb")
dobm=resample(dobm,s,method="ngb")
lbbm=resample(lbbm,s,method="ngb")
Ppbm=resample(Ppbm,s,method="ngb")

#Plot
evir_now=stack(depthbm,landDbm,cvbm,dobm,salbm,tmeanbm,trangebm,lbbm,P
pbm)
plot(evir_now)
names(evir_now)=c("depthbm","landDbm","cvbm","dobm","salbm","tmeanbm",
"trangebm","lbbm","Ppbm")
plot(evir_now)

##Future environmental factors
##2050rcp26----
#4future
cv_5026=readGDAL("environment/future/2050RCP26/Current_Velocity_Mean.t
if")
sal_5026=readGDAL("environment/future/2050RCP26/Salinity_Mean.tif")
tmean_5026=readGDAL("environment/future/2050RCP26/Temperature_Mean.tif
")
trange_5026=readGDAL("environment/future/2050RCP26/Temperature_Range.t
if")
#5present
depth_5026=readGDAL("environment/present/Depth2.tif")
landD_5026=readGDAL("environment/present/Land_Distance2.tif")
do_5026=readGDAL("environment/present/Dissolved_oxygen.tif")
lb_5026=readGDAL("environment/Present/Light_bottom.tif")
Pp_5026=readGDAL("environment/present/Primary_productivity.tif")

#4future#raster
cv_5026=raster(cv_5026)
sal_5026=raster(sal_5026)
tmean_5026=raster(tmean_5026)
trange_5026=raster(trange_5026)
#5present
depth_5026=raster(depth_5026)
landD_5026=raster(landD_5026)
do_5026=raster(do_5026)
lb_5026=raster(lb_5026)
Pp_5026=raster(Pp_5026)
```

```
##Define a scope
e<-extent(90 , 140 , -11, 15 )
s<-raster(e, nrows=312, ncols=600)
#crop by e
depth_5026=crop(depth_5026,e)
landD_5026=crop(landD_5026,e)
cv_5026=crop(cv_5026,e)
sal_5026=crop(sal_5026,e)
tmean_5026=crop(tmean_5026,e)
trange_5026=crop(trange_5026,e)
do_5026=crop(do_5026,e)
lb_5026=crop(lb_5026,e)
Pp_5026=crop(Pp_5026,e)

#env=reseample(env,s,method="ngb)
#Transfer values between mismatched grid objects (based on origin and
resolution)
#4future
cv_5026=resample(cv_5026,s,method="ngb")
sal_5026=resample(sal_5026,s,method="ngb")
tmean_5026=resample(tmean_5026,s,method="ngb")
trange_5026=resample(trange_5026,s,method="ngb")
#5present
depth_5026=resample(depth_5026,s,method="ngb")
landD_5026=resample(landD_5026,s,method="ngb")
do_5026=resample(do_5026,s,method="ngb")
lb_5026=resample(lb_5026,s,method="ngb")
Pp_5026=resample(Pp_5026,s,method="ngb")

#4future+5present
evir_5026=stack(cv_5026,sal_5026,tmean_5026,trange_5026,depth_5026,lan
dD_5026,do_5026,lb_5026,Pp_5026)
names(evir_5026)=c("cvbm","salbm","tmeanbm","trangebm","depthbm","land
Dbm","dobm","lbbm","Ppbm")
plot(evir_5026)

##2050rcp85----
#4future
cv_5085=readGDAL("environment/future/2050RCP85/Current_Velocity_Mean.t
if")
sal_5085=readGDAL("environment/future/2050RCP85/Salinity_Mean.tif")
tmean_5085=readGDAL("environment/future/2050RCP85/Temperature_Mean.tif
")
```

```
trange_5085=readGDAL("environment/future/2050RCP85/Temperature_Range.t
if")
#5present
depth_5085=readGDAL("environment/present/Depth2.tif")
landD_5085=readGDAL("environment/present/Land_Distance2.tif")
do_5085=readGDAL("environment/present/Dissolved_oxygen.tif")
lb_5085=readGDAL("environment/Present/Light_bottom.tif")
Pp_5085=readGDAL("environment/present/Primary_productivity.tif")

#4future
cv_5085=raster(cv_5085)
sal_5085=raster(sal_5085)
tmean_5085=raster(tmean_5085)
trange_5085=raster(trange_5085)
#5present
depth_5085=raster(depth_5085)
landD_5085=raster(landD_5085)
do_5085=raster(do_5085)
lb_5085=raster(lb_5085)
Pp_5085=raster(Pp_5085)

##Define a scope
e<-extent(90 , 140 , -11, 15 )
s<-raster(e, nrows=312, ncols=600)
#crop by e
depth_5085=crop(depth_5085,e)
landD_5085=crop(landD_5085,e)
cv_5085=crop(cv_5085,e)
sal_5085=crop(sal_5085,e)
tmean_5085=crop(tmean_5085,e)
trange_5085=crop(trange_5085,e)
do_5085=crop(do_5085,e)
lb_5085=crop(lb_5085,e)
Pp_5085=crop(Pp_5085,e)

#4future
cv_5085=resample(cv_5085,s,method="ngb")
sal_5085=resample(sal_5085,s,method="ngb")
tmean_5085 =resample(tmean_5085,s,method="ngb")
trange_5085=resample(trange_5085,s,method="ngb")
#5present
depth_5085=resample(depth_5085,s,method="ngb")
landD_5085=resample(landD_5085,s,method="ngb")
do_5085=resample(do_5085,s,method="ngb")
```

```
lb_5085=resample(lb_5085,s,method="ngb")
Pp_5085=resample(Pp_5085,s,method="ngb")

###4future+5present
evir_5085=stack(cv_5085,sal_5085,tmean_5085,trange_5085,depth_5085,lan
dD_5085,do_5085,lb_5085,Pp_5085)
names(evir_5085)=c("cvbm","salbm","tmeanbm","trangebm","depthbm","land
Dbm","dobm","lbbm","Ppbm")
plot(evir_5085)

##2100rcp26----
#4future
cv_0026=readGDAL("environment/future/2100RCP26/Current_Velocity_Mean.t
if")
sal_0026=readGDAL("environment/future/2100RCP26/Salinity_Mean.tif")
tmean_0026=readGDAL("environment/future/2100RCP26/Temperature_Mean.tif
")
trange_0026=readGDAL("environment/future/2100RCP26/Temperature_Range.t
if")
#5present
depth_0026=readGDAL("environment/present/Depth2.tif")
landD_0026=readGDAL("environment/present/Land_Distance2.tif")
do_0026=readGDAL("environment/present/Dissolved_oxygen.tif")
lb_0026=readGDAL("environment/Present/Light_bottom.tif")
Pp_0026=readGDAL("environment/present/Primary_productivity.tif")

#4future
cv_0026=raster(cv_0026)
sal_0026=raster(sal_0026)
tmean_0026=raster(tmean_0026)
trange_0026=raster(trange_0026)
#5present
depth_0026=raster(depth_0026)
landD_0026=raster(landD_0026)
do_0026=raster(do_0026)
lb_0026=raster(lb_0026)
Pp_0026=raster(Pp_0026)

##Define a scope
e<-extent(90 , 140 , -11, 15 )
s<-raster(e, nrows=312, ncols=600)
#crop by e
depth_0026=crop(depth_0026,e)
landD_0026=crop(landD_0026,e)
```

```
cv_0026=crop(cv_0026,e)
sal_0026=crop(sal_0026,e)
tmean_0026=crop(tmean_0026,e)
trange_0026=crop(trange_0026,e)
do_0026=crop(do_0026,e)
lb_0026=crop(lb_0026,e)
Pp_0026=crop(Pp_0026,e)

#4future
cv_0026=resample(cv_0026,s,method="ngb")
sal_0026=resample(sal_0026,s,method="ngb")
tmean_0026=resample(tmean_0026,s,method="ngb")
trange_0026=resample(trange_0026,s,method="ngb")
#5present
depth_0026=resample(depth_0026,s,method="ngb")
landD_0026=resample(landD_0026,s,method="ngb")
do_0026=resample(do_0026,s,method="ngb")
lb_0026=resample(lb_0026,s,method="ngb")
Pp_0026=resample(Pp_0026,s,method="ngb")

#4future+5present
evir_0026=stack(cv_0026,sal_0026,tmean_0026,trange_0026,depth_0026,lan
dD_0026,do_0026,lb_0026,Pp_0026)
names(evir_0026)=c("cvbm","salbm","tmeanbm","trangebm","depthbm","land
Dbm","dobm","lbbm","Ppbm")
plot(evir_0026)

##2100rcp85----
#4future
cv_0085=readGDAL("environment/future/2100RCP85/Current_Velocity_Mean.t
if")
sal_0085=readGDAL("environment/future/2100RCP85/Salinity_Mean.tif")
tmean_0085=readGDAL("environment/future/2100RCP85/Temperature_Mean.tif
")
trange_0085=readGDAL("environment/future/2100RCP85/Temperature_Range.t
if")
#5present
depth_0085=readGDAL("environment/present/Depth2.tif")
landD_0085=readGDAL("environment/present/Land_Distance2.tif")
do_0085=readGDAL("environment/present/Dissolved_oxygen.tif")
lb_0085=readGDAL("environment/Present/Light_bottom.tif")
Pp_0085=readGDAL("environment/present/Primary_productivity.tif")

#4future
```

```
cv_0085=raster(cv_0085)
sal_0085=raster(sal_0085)
tmean_0085=raster(tmean_0085)
trange_0085=raster(trange_0085)
#5present
depth_0085=raster(depth_0085)
landD_0085=raster(landD_0085)
do_0085=raster(do_0085)
lb_0085=raster(lb_0085)
Pp_0085=raster(Pp_0085)

##Define a scope
e<-extent(90 , 140 , -11, 15 )
s<-raster(e, nrows=312, ncols=600)
#crop by e
depth_0085=crop(depth_0085,e)
landD_0085=crop(landD_0085,e)
cv_0085=crop(cv_0085,e)
sal_0085=crop(sal_0085,e)
tmean_0085=crop(tmean_0085,e)
trange_0085=crop(trange_0085,e)
do_0085=crop(do_0085,e)
lb_0085=crop(lb_0085,e)
Pp_0085=crop(Pp_0085,e)

#4future
cv_0085=resample(cv_0085,s,method="ngb")
sal_0085=resample(sal_0085,s,method="ngb")
tmean_0085=resample(tmean_0085,s,method="ngb")
trange_0085=resample(trange_0085,s,method="ngb")
#5present
depth_0085=resample(depth_0085,s,method="ngb")
landD_0085=resample(landD_0085,s,method="ngb")
do_0085=resample(do_0085,s,method="ngb")
lb_0085=resample(lb_0085,s,method="ngb")
Pp_0085=resample(Pp_0085,s,method="ngb")

#4future+5present
evir_0085=stack(cv_0085,sal_0085,tmean_0085,trange_0085,depth_0085,lan
dD_0085,do_0085,lb_0085,Pp_0085)
names(evir_0085)=c("cvbm","salbm","tmeanbm","trangebm","depthbm","land
Dbm","dobm","lbbm","Ppbm")
plot(evir_0085)
```

```
## 四Pearson correlation and VIF analysis of environmental factors----

# Extract environment variables for sampling points
env_jh <-
list(depthbm,landDbm,cvbm,salbm,tmeanbm,trangebm,dobm,lbbm,Ppbm)
env_name<-
c("depthbm","landDbm","cvbm","salbm","tmeanbm","trange","dobm","lbbm",
"Ppbm")
env_extract <- list()
for (i in 1:length(env_name)) {
  env_extract[[i]] <- raster::extract(raster::stack(env_jh[[i]]),
A51[1:2])
}
env_extract <- data.frame(na.omit(data.frame(env_extract)))
names(env_extract) <- env_name

present <- as.matrix(seq(from = 1.0, to = 1.0, length.out =
nrow(env_extract)))
env_extract1 <- cbind(present, env_extract)
#Analysis+Drawing
#pearson correlation analysis
env.cor <- round(cor(env_extract, method = "pearson"), 3)
library(ggcorrplot)
env.p <- round(cor_pmat(env_extract, method = "pearson"), 3)
library(corrplot)
par(mfrow = c(1, 1), mar = c(2, 2, 1, 1))
cor.plot <- corrplot(corr = env.cor, type = "upper", tl.pos = "tp",
tl.col = "black", tl.cex = 1.2, cl.cex = 1, p.mat = env.p, insig =
"label_sig", sig.level = c(.01, .05), pch.cex = 1.5, pch.col = "black",
order = "original")
cor.plot <- corrplot(
  corr = env.cor, type = "lower", add = TRUE, method = "number",
  tl.pos = "n", tl.col = "black", # tl.cex标签字体大小
  col = "black", tl.cex = 1.3, diag = FALSE, cl.pos = "n", pch.col =
"black", # cl.cex图例字体大小
  number.cex = 1, number.font = 1, order = "original"
)
#Scatter/collinearity
library(car)
scatterplotMatrix(env_extract, main = "correlation")

##（五） Biomod2----
##simulate---
## 1. Formatting Data----
```

```
COLU=rep(1)
#D1=data.frame(cbind(COLU,A51[2:3]))
D1=data.frame(cbind(COLU,A51))

DataSpecies <- D1
head(DataSpecies)
myRespName <- 'COLU'
myResp <- as.numeric(DataSpecies[, myRespName])
myRespXY <- DataSpecies[, c("lon", "lat")]
myExpl <- evir_now

D_COLU<-
  BIOMOD_FormatingData(  #Rearrange user input data to ensure they can be used in biomod2
    resp.var = myResp,        #Vector 1 represents existing data and constructs a species distribution model
    expl.var = myExpl,          #RasterStack，containing explanatory variables for building the model (environment layer)
    resp.xy = myRespXY,     #Distribution Point
    resp.name = myRespName, #Response variable name. Species name.
    PA.nb.rep = 1,
    PA.nb.absences = 200,   #Pseudo non distributed number of points selected repeatedly each time
    PA.strategy = 'random',
    na.rm = TRUE)

## 2. Defining Models Options using default options.----

myBiomodOption <- BIOMOD_ModelingOptions() #Single model option to parameterize and/or adjust biomod

## 3. Doing Modelisation

R_COLU <-
  BIOMOD_Modeling(
    D_COLU,
    models = c('GLM','GBM', 'GAM', 'CTA', 'ANN', 'SRE', 'FDA', 'RF', 'MAXENT.Phillips','MARS'),
    models.options = myBiomodOption,
    NbRunEval = 10,                       #Number of times to run the evaluation
    DataSplit = 80,                    #80% for calibration and 20% for prediction
```

```
    Prevalence = 0.5,
    VarImport = 3,                    #The number of permutations for
estimating the importance of variables
    models.eval.meth = c('TSS','ROC'),  #evaluating indicator
    SaveObj = TRUE,
    do.full.models = FALSE,
    rescal.all.models=TRUE,
    modeling.id = "test"
  )
R_COLU

##The Importance of Environmental Variables----
gx=get_variables_importance(R_COLU)
gx
write.csv(gx,file = "importance.csv")
gx<- read.csv("importance.csv",header=T,row.names = 1)
gx <- t(gx)
ncol(gx)

gxmean<-c()
gxsd<-c()
for (i in 1:ncol(gx)) {
  gxmean[i]<-mean(gx[,i])
  gxsd[i]<-sd(gx[,i])/sqrt(nrow(gx))
}

gxdata<-cbind(gxmean, gxsd)
gxdata <- data.frame(gxdata)
gxdata
gxfig<-gxdata[,(ncol(gxdata)-1):(ncol(gxdata))]
gxfig
write.csv(gxfig,file = "importance_plot.csv")

library(forcats)
library(ggplot2)
par(mar=c(5.1, 5.1, 1.6, 1.1))
gxdata <- data.frame(names =
c("depthbm","landDbm","cvbm","salbm","tmeanbm","trangebm","dobm","lbbm
","Ppbm"), gxfig[,1:2])
gxdata$names <- fct_relevel(gxdata$names,
c("depthbm","landDbm","cvbm","salbm","tmeanbm","trangebm","dobm","lbbm
","Ppbm")) #调整柱子位置
gxresult <- ggplot(gxdata,aes(x=names, y=gxmean,fill=factor(names))) +
  geom_bar(stat = "identity", position="dodge", width=0.5,color="black")
```

```
+
  ## Bar chart line color and fill color
  geom_errorbar(aes(ymin=gxmean-gxsd, ymax=gxmean+gxsd), width=0.1,
                color="blue",## Error bar color
                position=position_dodge(0.6))+
  theme_bw()+##Remove background color
  theme(legend.title                                              =
element_blank(),panel.grid=element_blank())+##The title of the legend
is blank, with grid lines removed
  guides(fill="none")+ylab("Variable  Importance")+xlab("")  +#Remove
Legend Horizontal and Vertical Coordinate Names

scale_y_continuous(expand=c(0,0),breaks=seq(0,1.5,0.1))+expand_limits(
y=0.53)+ #The second parameter of seq is the range that includes the
data
  theme(panel.border = element_blank(), axis.line = element_line(),
#Remove borders and only preserve the xy axis
        axis.line.y=element_line(linetype=1,color="black",size=1),
#Y-axis thickness
        axis.line.x=element_line(linetype=1,color="black",size=1),
#X-axis thickness
        plot.title = element_text(hjust = 0,size = 18, face = "bold"),
#Title position in the upper left corner
        axis.text=element_text(size=15),                  #Horizontal
and vertical scale font size
        axis.title.x =element_text(size=18,face = "bold"),
        axis.title.y=element_text(size=18,face = "bold")) #Horizontal
and vertical coordinate title font size
gxresult
gxdata


##model evaluation----
## Evaluation ----

E_COLU <- get_evaluations(R_COLU)

E_COLU
## TSS AUC----
tssdata<-E_COLU["TSS", "Testing.data" ,,,]
tssmean<-c()
tsssd<-c()
for (i in 1:nrow(tssdata)) {               #For loop finding mean and
standard error
```

```
  tssmean[i]<-mean(tssdata[i,])
  tsssd[i]<-sd(tssdata[i,])/sqrt(ncol(tssdata))
}
tssdata<-cbind(tssdata, tssmean, tsssd)
dim(tssdata)

tssfig<-tssdata[,(ncol(tssdata)-1):(ncol(tssdata))]      #The last two
columns of tssdata
tssfig
rownames(tssfig)[9]<-"Maxent"
tssfig
write.csv(tssfig,file = "TSSplot.csv")

##Bar chart+standard error
library(ggplot2)
par(mar=c(5.1, 5.1, 1.6, 1.1))
tssdata <- data.frame(names = c('GLM','GBM', 'GAM', 'CTA', 'ANN', 'SRE',
'FDA', 'RF', 'MAXENT','MARS')
                     , tssfig[,1:2])
TSSresult <- ggplot(tssdata,aes(x=names, y=tssmean,fill=factor(names)))
+
  geom_bar(stat      =      "identity",      position=position_dodge(),
width=0.6,color="black") +

  geom_errorbar(aes(ymin=tssmean-tsssd, ymax=tssmean+tsssd), width=0.2,
                color="blue",
                position=position_dodge(0.6))+
  theme_bw()+
  theme(legend.title = element_blank(),panel.grid=element_blank())+
  guides(fill="none")+ylab('TSS value')+xlab("") +

scale_y_continuous(expand=c(0,0),breaks=seq(0,1.5,0.2))+expand_limits(
y=1.05)+
  theme(panel.border = element_blank(), axis.line = element_line(),
        axis.line.y=element_line(linetype=1,color="black",size=1),
        axis.line.x=element_line(linetype=1,color="black",size=1),
        plot.title = element_text(hjust = 0,size = 18, face = "bold"),
        axis.text=element_text(size=12),
        axis.title.x =element_text(size=18,face = "bold"),
        axis.title.y=element_text(size=18,face = "bold"))+
  geom_hline(aes(yintercept=0.80),linetype=5,col="black",size=0.7)
TSSresult
```

```
##AUC
rocdata<-E_COLU["ROC", "Testing.data" ,,,]
rocmean<-c()
rocsd<-c()
for (i in 1:nrow(rocdata)) {
  rocmean[i]<-mean(rocdata[i,])
  rocsd[i]<-sd(rocdata[i,])/sqrt(ncol(rocdata))
}
rocdata<- cbind(rocdata, rocmean, rocsd)
dim(rocdata)

rocfig<-rocdata[,(ncol(rocdata)-1):(ncol(rocdata))]
rocfig
rownames(rocfig)[9]<-"Maxent"
rocfig
write.csv(rocfig,file = "AUCplot.csv")

##Bar chart+standard error
library(cowplot)
par(mar=c(5.1, 5.1, 1.6, 1.1))
rocdata <- data.frame(names = c('GLM','GBM', 'GAM', 'CTA', 'ANN', 'SRE',
'FDA', 'RF', 'MAXENT','MARS')
                     , rocfig[,1:2])
AUCresult <- ggplot(rocdata,aes(x=names, y=rocmean,fill=factor(names)))
+
  geom_bar(stat      =      "identity",      position=position_dodge(),
width=0.6,color="black") +
  ## Bar chart line color and fill color
  geom_errorbar(aes(ymin=rocmean-rocsd, ymax=rocmean+rocsd), width=0.2,
              color="blue", ## Error bar color
              position=position_dodge(0.6))+
  theme_bw()+
  theme(legend.title = element_blank(),panel.grid=element_blank())+
  guides(fill="none")+ylab('AUC value')+xlab("") +

scale_y_continuous(expand=c(0,0),breaks=seq(0,1.5,0.2))+expand_limits(
y=1.05) +
  theme(panel.border = element_blank(), axis.line = element_line(),
        axis.line.y=element_line(linetype=1,color="black",size=1),
        axis.line.x=element_line(linetype=1,color="black",size=1),
        plot.title = element_text(hjust = 0,size = 18, face = "bold"),
        axis.text=element_text(size=12),
        axis.title.x =element_text(size=18,face = "bold"),
        axis.title.y=element_text(size=18,face = "bold"))+
```

```
  geom_hline(aes(yintercept=0.90),linetype=5,col="black",size=0.7)
AUCresult
results<-cbind(tssfig, rocfig)
results
write.csv(results, file = 'TSS and AUC')

## response curv----
#mySREs<-BIOMOD_LoadModels(R_COLU,  models  =  'SRE')   #Load  a  single
model
myGLMS<-BIOMOD_LoadModels(R_COLU, models = 'GLM')
myGAMS<-BIOMOD_LoadModels(R_COLU, models = 'GAM')
myCTAS<-BIOMOD_LoadModels(R_COLU, models = 'CTA')
myANNS<-BIOMOD_LoadModels(R_COLU, models = 'ANN')
myFDAS<-BIOMOD_LoadModels(R_COLU, models = 'FDA')
myRFS <-BIOMOD_LoadModels(R_COLU, models = 'RF')
myGBMS<-BIOMOD_LoadModels(R_COLU, models = 'GBM')
myMARS<-BIOMOD_LoadModels(R_COLU, models = 'MARS')

mybio1=BIOMOD_LoadModels(R_COLU,expl.var.names="bio1")
##' 4.2 plot 2D response plots
RC_GLM <-
  response.plot2(
    models = myGLMS,
    Data = get_formal_data(R_COLU, 'expl.var'),
    show.variables = get_formal_data(R_COLU,'expl.var.names'),
    do.bivariate = FALSE,
    fixed.var.metric = 'mean',
    ImageSize = 320,
    legend = TRUE,
    data_species = get_formal_data(R_COLU, 'resp.var')
  )
RC_GAM <-
  response.plot2(
    models = myGAMS,
    Data = get_formal_data(R_COLU, 'expl.var'),
    show.variables = get_formal_data(R_COLU,'expl.var.names'),
    do.bivariate = FALSE,
    fixed.var.metric = 'mean',
    ImageSize = 320,
    legend = TRUE,
    data_species = get_formal_data(R_COLU, 'resp.var')
  )
RC_GBM <-
  response.plot2(
```

```
    models = myGBMS,
    Data = get_formal_data(R_COLU, 'expl.var'),
    show.variables = get_formal_data(R_COLU,'expl.var.names'),
    do.bivariate = FALSE,
    fixed.var.metric = 'mean',
    ImageSize = 320,
    legend = FALSE,
    data_species = get_formal_data(R_COLU, 'resp.var')
  )

RC_ANN <-
  response.plot2(
    models = myANNS,
    Data = get_formal_data(R_COLU, 'expl.var'),
    show.variables = get_formal_data(R_COLU,'expl.var.names'),
    do.bivariate = FALSE,
    fixed.var.metric = 'mean',
    ImageSize = 320,
    legend = TRUE,
    data_species = get_formal_data(R_COLU, 'resp.var')
  )
RC_CTA <-
  response.plot2(
    models = myCTAS,
    Data = get_formal_data(R_COLU, 'expl.var'),
    show.variables = get_formal_data(R_COLU,'expl.var.names'),
    do.bivariate = FALSE,
    fixed.var.metric = 'mean',
    ImageSize = 320,
    legend = TRUE,
    data_species = get_formal_data(R_COLU, 'resp.var')
  )
##' 4.2 plot 3D response plots

RC_FDA <-
  response.plot2(
    models = myFDAS,
    Data = get_formal_data(R_COLU, 'expl.var'),
    show.variables = get_formal_data(R_COLU,'expl.var.names'),
    do.bivariate = FALSE,
    fixed.var.metric = 'mean',
    ImageSize = 320,
    legend = TRUE,
    data_species = get_formal_data(R_COLU, 'resp.var')
```

```
  )
RC_RF <-
  response.plot2(
    models = myRFS,
    Data = get_formal_data(R_COLU, 'expl.var'),
    show.variables = get_formal_data(R_COLU,'expl.var.names'),
    do.bivariate = FALSE,
    fixed.var.metric = 'mean',
    ImageSize = 320,
    legend = TRUE,
    data_species = get_formal_data(R_COLU, 'resp.var')
  )
RC_MARS<-
  response.plot2(
    models = myMARS,
    Data = get_formal_data(R_COLU, 'expl.var'),
    show.variables = get_formal_data(R_COLU,'expl.var.names'),
    do.bivariate = FALSE,
    fixed.var.metric = 'mean',
    ImageSize = 320,
    legend = TRUE,
    data_species = get_formal_data(R_COLU, 'resp.var')
  )
#Expl. val is the horizontal axis (variable), and pred. val is the vertical axis (probability)

rp.gg.theme =theme(legend.title = element_blank(),legend.position="none",plot.title = element_text(hjust = 0.5),panel.grid.major =element_blank(), panel.grid.minor = element_blank(),panel.background = element_blank(),axis.line = element_line(colour = "black"))

GLMdepthbm=filter(RC_GLM, expl.name =="depthbm")
GLMlandDbm=filter(RC_GLM, expl.name =="landDbm")
GLMcvbm=filter(RC_GLM, expl.name =="cvbm")
GLMsalbm=filter(RC_GLM, expl.name =="salbm")
GLMtmeanbm=filter(RC_GLM, expl.name =="tmeanbm")
GLMtrangebm=filter(RC_GLM, expl.name =="trangebm")


GAMdepthbm=filter(RC_GAM, expl.name =="depthbm")
GAMlandDbm=filter(RC_GAM, expl.name =="landDbm")
GAMcvbm=filter(RC_GAM, expl.name =="cvbm")
GAMsalbm=filter(RC_GAM, expl.name =="salbm")
```

```
GAMtmeanbm=filter(RC_GAM, expl.name =="tmeanbm")
GAMtrangebm=filter(RC_GAM, expl.name =="trangebm")


GBMdepthbm=filter(RC_GBM, expl.name =="depthbm")
GBMlandDbm=filter(RC_GBM, expl.name =="landDbm")
GBMcvbm=filter(RC_GBM, expl.name =="cvbm")
GBMsalbm=filter(RC_GBM, expl.name =="salbm")
GBMtmeanbm=filter(RC_GBM, expl.name =="tmeanbm")
GBMtrangebm=filter(RC_GBM, expl.name =="trangebm")


ANNdepthbm=filter(RC_ANN, expl.name =="depthbm")
ANNlandDbm=filter(RC_ANN, expl.name =="landDbm")
ANNcvbm=filter(RC_ANN, expl.name =="cvbm")
ANNsalbm=filter(RC_ANN, expl.name =="salbm")
ANNtmeanbm=filter(RC_ANN, expl.name =="tmeanbm")
ANNtrangebm=filter(RC_ANN, expl.name =="trangebm")


CTAdepthbm=filter(RC_CTA, expl.name =="depthbm")
CTAlandDbm=filter(RC_CTA, expl.name =="landDbm")
CTAcvbm=filter(RC_CTA, expl.name =="cvbm")
CTAsalbm=filter(RC_CTA, expl.name =="salbm")
CTAtmeanbm=filter(RC_CTA, expl.name =="tmeanbm")
CTAtrangebm=filter(RC_CTA, expl.name =="trangebm")



RFdepthbm=filter(RC_RF, expl.name =="depthbm")
RFlandDbm=filter(RC_RF, expl.name =="landDbm")
RFcvbm=filter(RC_RF, expl.name =="cvbm")
RFsalbm=filter(RC_RF, expl.name =="salbm")
RFtmeanbm=filter(RC_RF, expl.name =="tmeanbm")
RFtrangebm=filter(RC_RF, expl.name =="trangebm")


MARSdepthbm=filter(RC_MARS, expl.name =="depthbm")
MARSlandDbm=filter(RC_MARS, expl.name =="landDbm")
MARScvbm=filter(RC_MARS, expl.name =="cvbm")
MARSsalbm=filter(RC_MARS, expl.name =="salbm")
MARStmeanbm=filter(RC_MARS, expl.name =="tmeanbm")
MARStrangebm=filter(RC_MARS, expl.name =="trangebm")
```

```
FDAdepthbm=filter(RC_FDA, expl.name =="depthbm")
FDAlandDbm=filter(RC_FDA, expl.name =="landDbm")
FDAcvbm=filter(RC_FDA, expl.name =="cvbm")
FDAsalbm=filter(RC_FDA, expl.name =="salbm")
FDAtmeanbm=filter(RC_FDA, expl.name =="tmeanbm")
FDAtrangebm=filter(RC_FDA, expl.name =="trangebm")


#1
glmdepthbm=ggplot(GLMdepthbm,aes(x = expl.val, y = pred.val, group=pred.name , fill=F))+ggtitle("GLM") + geom_line() + ylab("") + xlab("") + rp.gg.theme
glmlandDbm=ggplot(GLMlandDbm,aes(x = expl.val, y = pred.val, group=pred.name , fill=F))+ggtitle("GLM") + geom_line() + ylab("") + xlab("") + rp.gg.theme
glmcvbm=ggplot(GLMcvbm,aes(x = expl.val, y = pred.val, group=pred.name , fill=F))+ggtitle("GLM") + geom_line() + ylab("") + xlab("") + rp.gg.theme
glmsalbm=ggplot(GLMsalbm,aes(x = expl.val, y = pred.val, group=pred.name , fill=F))+ggtitle("GLM") + geom_line() + ylab("") + xlab("") + rp.gg.theme
glmtmeanbm=ggplot(GLMtmeanbm,aes(x = expl.val, y = pred.val, group=pred.name , fill=F))+ggtitle("GLM") + geom_line() + ylab("") + xlab("") + rp.gg.theme
glmtrangebm=ggplot(GLMtrangebm,aes(x = expl.val, y = pred.val, group=pred.name , fill=F))+ggtitle("GLM") + geom_line() + ylab("") + xlab("") + rp.gg.theme


#2
gbmdepthbm=ggplot(GBMdepthbm,aes(x = expl.val, y = pred.val, group=pred.name , fill=F))+ggtitle("GBM") + geom_line() + ylab("") + xlab("") + rp.gg.theme
gbmlandDbm=ggplot(GBMlandDbm,aes(x = expl.val, y = pred.val, group=pred.name , fill=F))+ggtitle("GBM") + geom_line() + ylab("") + xlab("") + rp.gg.theme
gbmcvbm=ggplot(GBMcvbm,aes(x = expl.val, y = pred.val, group=pred.name , fill=F))+ggtitle("GBM") + geom_line() + ylab("") + xlab("") + rp.gg.theme
gbmsalbm=ggplot(GBMsalbm,aes(x = expl.val, y = pred.val, group=pred.name , fill=F))+ggtitle("GBM") + geom_line() + ylab("") + xlab("") + rp.gg.theme
gbmtmeanbm=ggplot(GBMtmeanbm,aes(x = expl.val, y = pred.val,
```

```
group=pred.name , fill=F))+ggtitle("GBM") + geom_line() + ylab("") + xlab("") + rp.gg.theme
gbmtrangebm=ggplot(GBMtrangebm,aes(x = expl.val, y = pred.val, group=pred.name , fill=F))+ggtitle("GBM") + geom_line() + ylab("") + xlab("") + rp.gg.theme


#3
gamdepthbm=ggplot(GAMdepthbm,aes(x = expl.val, y = pred.val, group=pred.name , fill=F))+ggtitle("GAM") + geom_line() + ylab("") + xlab("") + rp.gg.theme
gamlandDbm=ggplot(GAMlandDbm,aes(x = expl.val, y = pred.val, group=pred.name , fill=F))+ggtitle("GAM") + geom_line() + ylab("") + xlab("") + rp.gg.theme
gamcvbm=ggplot(GAMcvbm,aes(x = expl.val, y = pred.val, group=pred.name , fill=F))+ggtitle("GAM") + geom_line() + ylab("") + xlab("") + rp.gg.theme
gamsalbm=ggplot(GAMsalbm,aes(x = expl.val, y = pred.val, group=pred.name , fill=F))+ggtitle("GAM") + geom_line() + ylab("") + xlab("") + rp.gg.theme
gamtmeanbm=ggplot(GAMtmeanbm,aes(x = expl.val, y = pred.val, group=pred.name , fill=F))+ggtitle("GAM") + geom_line() + ylab("") + xlab("") + rp.gg.theme
gamtrangebm=ggplot(GAMtrangebm,aes(x = expl.val, y = pred.val, group=pred.name , fill=F))+ggtitle("GAM") + geom_line() + ylab("") + xlab("") + rp.gg.theme


#4
anndepthbm=ggplot(ANNdepthbm,aes(x = expl.val, y = pred.val, group=pred.name , fill=F))+ggtitle("ANN") + geom_line() + ylab("") + xlab("") + rp.gg.theme
annlandDbm=ggplot(ANNlandDbm,aes(x = expl.val, y = pred.val, group=pred.name , fill=F))+ggtitle("ANN") + geom_line() + ylab("") + xlab("") + rp.gg.theme
anncvbm=ggplot(ANNcvbm,aes(x = expl.val, y = pred.val, group=pred.name , fill=F))+ggtitle("ANN") + geom_line() + ylab("") + xlab("") + rp.gg.theme
annsalbm=ggplot(ANNsalbm,aes(x = expl.val, y = pred.val, group=pred.name , fill=F))+ggtitle("ANN") + geom_line() + ylab("") + xlab("") + rp.gg.theme
anntmeanbm=ggplot(ANNtmeanbm,aes(x = expl.val, y = pred.val, group=pred.name , fill=F))+ggtitle("ANN") + geom_line() + ylab("") + xlab("") + rp.gg.theme
```

```
anntrangebm=ggplot(ANNtrangebm,aes(x = expl.val, y = pred.val, group=pred.name , fill=F))+ggtitle("ANN") + geom_line() + ylab("") + xlab("") + rp.gg.theme


#5
ctadepthbm=ggplot(CTAdepthbm,aes(x = expl.val, y = pred.val, group=pred.name, fill=F))+ggtitle("CTA") + geom_line() + ylab("") + xlab("") + rp.gg.theme
ctalandDbm=ggplot(CTAlandDbm,aes(x = expl.val, y = pred.val, group=pred.name, fill=F))+ggtitle("CTA") + geom_line() + ylab("") + xlab("") + rp.gg.theme
ctacvbm=ggplot(CTAcvbm,aes(x = expl.val, y = pred.val, group=pred.name, fill=F))+ggtitle("CTA") + geom_line() + ylab("") + xlab("") + rp.gg.theme
ctasalbm=ggplot(CTAsalbm,aes(x = expl.val, y = pred.val, group=pred.name , fill=F))+ggtitle("CTA") + geom_line() + ylab("") + xlab("") + rp.gg.theme
ctatmeanbm=ggplot(CTAtmeanbm,aes(x = expl.val, y = pred.val, group=pred.name , fill=F))+ggtitle("CTA") + geom_line() + ylab("") + xlab("") + rp.gg.theme
ctatrangebm=ggplot(CTAtrangebm,aes(x = expl.val, y = pred.val, group=pred.name , fill=F))+ggtitle("CTA") + geom_line() + ylab("") + xlab("") + rp.gg.theme


#6
fdadepthbm=ggplot(FDAdepthbm,aes(x = expl.val, y = pred.val, group=pred.name , fill=F))+ggtitle("FDA") + geom_line() + ylab("") + xlab("") + rp.gg.theme
fdalandDbm=ggplot(FDAlandDbm,aes(x = expl.val, y = pred.val, group=pred.name , fill=F))+ggtitle("FDA") + geom_line() + ylab("") + xlab("") + rp.gg.theme
fdacvbm=ggplot(FDAcvbm,aes(x = expl.val, y = pred.val, group=pred.name , fill=F))+ggtitle("FDA") + geom_line() + ylab("") + xlab("") + rp.gg.theme
fdasalbm=ggplot(FDAsalbm,aes(x = expl.val, y = pred.val, group=pred.name , fill=F))+ggtitle("FDA") + geom_line() + ylab("") + xlab("") + rp.gg.theme
fdatmeanbm=ggplot(FDAtmeanbm,aes(x = expl.val, y = pred.val, group=pred.name , fill=F))+ggtitle("FDA") + geom_line() + ylab("") + xlab("") + rp.gg.theme
fdatrangebm=ggplot(FDAtrangebm,aes(x = expl.val, y = pred.val, group=pred.name , fill=F))+ggtitle("FDA") + geom_line() + ylab("") +
```

```
xlab("") + rp.gg.theme


#7
rfdepthbm=ggplot(RFdepthbm,aes(x = expl.val, y = pred.val, group=pred.name , fill=F))+ggtitle("RF") + geom_line() + ylab("") + xlab("") + rp.gg.theme
rflandDbm=ggplot(RFlandDbm,aes(x = expl.val, y = pred.val, group=pred.name , fill=F))+ggtitle("RF") + geom_line() + ylab("") + xlab("") + rp.gg.theme
rfcvbm=ggplot(RFcvbm,aes(x = expl.val, y = pred.val, group=pred.name , fill=F))+ggtitle("RF") + geom_line() + ylab("") + xlab("") + rp.gg.theme
rfsalbm=ggplot(RFsalbm,aes(x = expl.val, y = pred.val, group=pred.name , fill=F))+ggtitle("RF") + geom_line() + ylab("") + xlab("") + rp.gg.theme
rftmeanbm=ggplot(RFtmeanbm,aes(x = expl.val, y = pred.val, group=pred.name , fill=F))+ggtitle("RF") + geom_line() + ylab("") + xlab("") + rp.gg.theme
rftrangebm=ggplot(RFtrangebm,aes(x = expl.val, y = pred.val, group=pred.name , fill=F))+ggtitle("RF") + geom_line() + ylab("") + xlab("") + rp.gg.theme



#9
marsdepthbm=ggplot(MARSdepthbm,aes(x = expl.val, y = pred.val, group=pred.name , fill=F))+ggtitle("MARS") + geom_line() + ylab("") + xlab("") + rp.gg.theme
marslandDbm=ggplot(MARSlandDbm,aes(x = expl.val, y = pred.val, group=pred.name , fill=F))+ggtitle("MARS") + geom_line() + ylab("") + xlab("") + rp.gg.theme
marscvbm=ggplot(MARScvbm,aes(x = expl.val, y = pred.val, group=pred.name , fill=F))+ggtitle("MARS") + geom_line() + ylab("") + xlab("") + rp.gg.theme
marsalbm=ggplot(MARSsalbm,aes(x = expl.val, y = pred.val, group=pred.name, fill=F))+ggtitle("MARS") + geom_line() + ylab("") + xlab("") + rp.gg.theme
marstmeanbm=ggplot(MARStmeanbm,aes(x = expl.val, y = pred.val, group=pred.name , fill=F))+ggtitle("MARS") + geom_line() + ylab("") + xlab("") + rp.gg.theme
marstrangebm=ggplot(MARStrangebm,aes(x = expl.val, y = pred.val, group=pred.name , fill=F))+ggtitle("MARS") + geom_line() + ylab("") + xlab("") + rp.gg.theme
```

```
##Ggdraw() - Initialize a drawing panel, draw_ Plot() - Draw the graph
in the initialized drawing panel
par(mfrow=c(3,1),mar=c(2,2,2,2))
RC_depthbm=ggdraw()+draw_plot(glmdepthbm,0,
0, .33, .33)+draw_plot(gamdepthbm,0, .33, .33, .33)+draw_plot(gbmdepth
bm,.66, 0, .33, .33)+draw_plot(ctadepthbm,.33,
0, .33, .33)+draw_plot(anndepthbm,.33, .33, .33, .33)+draw_plot(rfdept
hbm,.33, .66, .33, .33)+draw_plot(fdadepthbm,.66, .66, .33, .33)+draw_
plot(marsdepthbm,.66, .33, .33, .33)
RC_landDbm=ggdraw()+draw_plot(glmlandDbm,0,
0, .33, .33)+draw_plot(gamlandDbm,0, .33, .33, .33)+draw_plot(gbmlandD
bm,.66, 0, .33, .33)+draw_plot(ctalandDbm,.33,
0, .33, .33)+draw_plot(annlandDbm,.33, .33, .33, .33)+draw_plot(rfland
Dbm,.33, .66, .33, .33)+draw_plot(fdalandDbm,.66, .66, .33, .33)+draw_
plot(marslandDbm,.66, .33, .33, .33)
RC_cvbm=ggdraw()+draw_plot(glmcvbm,0,
0, .33, .33)+draw_plot(gamcvbm,0, .33, .33, .33)+draw_plot(gbmcvbm,.66,
0, .33, .33)+draw_plot(ctacvbm,.33,
0, .33, .33)+draw_plot(anncvbm,.33, .33, .33, .33)+draw_plot(rfcvbm,.3
3, .66, .33, .33)+draw_plot(fdacvbm,.66, .66, .33, .33)+draw_plot(mars
cvbm,.66, .33, .33, .33)
RC_salbm=ggdraw()+draw_plot(glmsalbm,0,
0, .33, .33)+draw_plot(gamsalbm,0, .33, .33, .33)+draw_plot(gbmsalbm,.
66, 0, .33, .33)+draw_plot(ctasalbm,.33,
0, .33, .33)+draw_plot(annsalbm,.33, .33, .33, .33)+draw_plot(rfsalbm,
.33, .66, .33, .33)+draw_plot(fdasalbm,.66, .66, .33, .33)+draw_plot(m
arsalbm,.66, .33, .33, .33)
RC_tmeanbm=ggdraw()+draw_plot(glmtmeanbm,0,
0, .33, .33)+draw_plot(gamtmeanbm,0, .33, .33, .33)+draw_plot(gbmtmean
bm,.66, 0, .33, .33)+draw_plot(ctatmeanbm,.33,
0, .33, .33)+draw_plot(anntmeanbm,.33, .33, .33, .33)+draw_plot(rftmea
nbm,.33, .66, .33, .33)+draw_plot(fdatmeanbm,.66, .66, .33, .33)+draw_
plot(marstmeanbm,.66, .33, .33, .33)
RC_trangebm=ggdraw()+draw_plot(glmtrangebm,0,
0, .33, .33)+draw_plot(gamtrangebm,0, .33, .33, .33)+draw_plot(gbmtran
gebm,.66, 0, .33, .33)+draw_plot(ctatrangebm,.33,
0, .33, .33)+draw_plot(anntrangebm,.33, .33, .33, .33)+draw_plot(rftra
ngebm,.33, .66, .33, .33)+draw_plot(fdatrangebm,.66, .66, .33, .33)+dr
aw_plot(marstrangebm,.66, .33, .33, .33)



##Project----
##Projection on current environmental conditions----
```

```
#Predict potential distribution
P_COLU  <-  BIOMOD_Projection(modeling.output  =  R_COLU,       ##BIOMOD_
Modeling run results
                            new.env              =              myExpl,
#Environment Collection
                            proj.name = 'current',        #Create a new
folder with this name
                            selected.models = 'all',
                            binary.meth = 'TSS',          #Probability
threshold for selecting the maximum TSS value
                            compress = FALSE,              #compressed
format
                            build.clamping.mask = FALSE)
#Create and evaluate a set of ensemble models and predictions
EM_COLU <- BIOMOD_EnsembleModeling( modeling.output = R_COLU,
                                    chosen.models = 'all',
                                    em.by = 'all',
                                    eval.metric       =       c('TSS'),
#Constructing evaluation indicators for ensemble models
                                    eval.metric.quality.threshold     =
c(0.7), ##Models below this value will be excluded from integrated model
construction
                                    models.eval.meth    =    c('TSS'),
#Evaluation indicators of ensemble models
                                    prob.mean          =          TRUE,
#Estimating the average probability of different predictions
                                    prob.cv          =           FALSE,
#Estimating the coefficient of variation for different predictions
                                    prob.ci          =           FALSE,
#Estimate the confidence interval around the mean probability
                                    prob.ci.alpha        =        0.05,
#Estimate the significance level of the confidence interval, default to
0.05      prob.median = FALSE,
                                    committee.averaging = FALSE,
                                    prob.mean.weight = FALSE,
                                    prob.mean.weight.decay            =
'proportional' )
#Construct a set of projections of species in space and time
EMP_COLU=BIOMOD_EnsembleForecasting( projection.output = P_COLU,
                                     binary.meth = 'TSS',
                                     EM.output = EM_COLU)
```

```
current_COLU<-
raster::stack("D:/SDM_Tridacna/Standardization/step5_future_prediction
/TS/COLU/proj_current/proj_current_COLU_ensemble_TSSbin.grd")
plot(current_COLU)


##  Projection on future environemental conditions----

##projection5026
P_5026 <- BIOMOD_Projection(modeling.output = R_COLU,
                            new.env = evir_5026,
                            proj.name = '5026',
                            selected.models = 'all',
                            binary.meth = 'TSS',
                            compress = FALSE,
                            build.clamping.mask = TRUE)


##projection5085
P_5085 <- BIOMOD_Projection(modeling.output = R_COLU,
                            new.env = evir_5085,
                            proj.name = '5085',
                            selected.models = 'all',
                            binary.meth = 'TSS',
                            compress = FALSE,
                            build.clamping.mask = TRUE)
##projection0026
P_0026 <- BIOMOD_Projection(modeling.output = R_COLU,
                            new.env = evir_0026,
                            proj.name = '0026',
                            selected.models = 'all',
                            binary.meth = 'TSS',
                            compress = FALSE,
                            build.clamping.mask = TRUE)

##projection0085
P_0085 <- BIOMOD_Projection(modeling.output = R_COLU,
                            new.env = evir_0085,
                            proj.name = '0085',
                            selected.models = 'all',
                            binary.meth = 'TSS',
                            compress = FALSE,
                            build.clamping.mask = TRUE)
```

```
##  EnsembleForecasting on future environemental conditions----
EMF_5026=BIOMOD_EnsembleForecasting( projection.output = P_5026,
                                     binary.meth = 'TSS',
                                     EM.output = EM_COLU)

EMF_5085=BIOMOD_EnsembleForecasting( projection.output = P_5085,
                                     binary.meth = 'TSS',
                                     EM.output = EM_COLU)

EMF_0026=BIOMOD_EnsembleForecasting( projection.output = P_0026,
                                     binary.meth = 'TSS',
                                     EM.output = EM_COLU)

EMF_0085=BIOMOD_EnsembleForecasting( projection.output = P_0085,
                                     binary.meth = 'TSS',
                                     EM.output = EM_COLU)

##  PLOT----
par(mfcol=c(1,1),mar=c(1,1,1,1),oma=c(2,2,2,2))
###binary
OPB_COLU<-
raster::stack("D:/SDM_Tridacna/Standardization/step5_future_prediction
/TS/COLU/proj_current/proj_current_COLU_ensemble_TSSbin.grd")
OPB_5026                                                             <-
raster::stack("COLU/proj_5026/proj_5026_COLU_ensemble_TSSbin.grd")
OPB_5085                                                             <-
raster::stack("COLU/proj_5085/proj_5085_COLU_ensemble_TSSbin.grd")
OPB_0026                                                             <-
raster::stack("COLU/proj_0026/proj_0026_COLU_ensemble_TSSbin.grd")
OPB_0085                                                             <-
raster::stack("COLU/proj_0085/proj_0085_COLU_ensemble_TSSbin.grd")
plot(OPB_COLU,colNA=("gray70"))
plot(OPB_5026,colNA=("gray70"))
plot(OPB_5085,colNA=("gray70"))
plot(OPB_0026,colNA=("gray70"))
plot(OPB_0085,colNA=("gray70"))

plot(OPB_COLU,colNA=("#91522d"))
plot(OPB_5026,colNA=("#91522d"))
plot(OPB_5085,colNA=("#91522d"))
plot(OPB_0026,colNA=("#91522d"))
plot(OPB_0085,colNA=("#91522d"))
###normal
```

```
OPN_COLU<-
raster::stack("COLU/proj_current/individual_projections/COLU_EMmeanByTSS_mergedAlgo_mergedRun_mergedData.grd")
OPN_5026 <-
raster::stack("COLU/proj_5026/individual_projections/COLU_EMmeanByTSS_mergedAlgo_mergedRun_mergedData.grd")
OPN_5085 <-
raster::stack("COLU/proj_5085/individual_projections/COLU_EMmeanByTSS_mergedAlgo_mergedRun_mergedData.grd")
OPN_0026 <-
raster::stack("COLU/proj_0026/individual_projections/COLU_EMmeanByTSS_mergedAlgo_mergedRun_mergedData.grd")
OPN_0085 <-
raster::stack("COLU/proj_0085/individual_projections/COLU_EMmeanByTSS_mergedAlgo_mergedRun_mergedData.grd")
plot(OPN_COLU,colNA=("gray70"),legend=F,xlab="Longitude(° )",ylab="Laitude(° )")
plot(OPN_5026,colNA=("gray70"),legend=F,xlab="Longitude(° )",ylab="Laitude(° )")
plot(OPN_5085,colNA=("gray70"),legend=F,xlab="Longitude(° )",ylab="Laitude(° )")
plot(OPN_0026,colNA=("gray70"),legend=F,xlab="Longitude(° )",ylab="Laitude(° )")
plot(OPN_0085,colNA=("gray70"),legend=F,xlab="Longitude(° )",ylab="Laitude(° )")


plot(OPN_COLU,colNA=("#91522d"),legend=F,xlab="Longitude(° )",ylab="Laitude(° )")
plot(OPN_5026,colNA=("#91522d"),legend=F,xlab="Longitude(° )",ylab="Laitude(° )")
plot(OPN_5085,colNA=("#91522d"),legend=F,xlab="Longitude(° )",ylab="Laitude(° )")
plot(OPN_0026,colNA=("#91522d"),legend=F,xlab="Longitude(° )",ylab="Laitude(° )")
plot(OPN_0085,colNA=("#91522d"),legend=F,xlab="Longitude(° )",ylab="Laitude(° )")
##  Rangeshift----
RSB_5026<- BIOMOD_RangeSize(CurrentPred=OPB_COLU,FutureProj=OPB_5026)
RSB_5085<- BIOMOD_RangeSize(CurrentPred=OPB_COLU,FutureProj=OPB_5085)
RSB_0026<- BIOMOD_RangeSize(CurrentPred=OPB_COLU,FutureProj=OPB_0026)
RSB_0085<- BIOMOD_RangeSize(CurrentPred=OPB_COLU,FutureProj=OPB_0085)
RSB_5026$Compt.By.Models
RSB_5085$Compt.By.Models
```

```
RSB_0026$Compt.By.Models
RSB_0085$Compt.By.Models

#Legend Position          Name              Color
par(mfrow=c(1,1),mai=c(0.3,0.3,0.3,0.3))

plot(RSB_5026$Diff.By.Pixel,col=c("blue","green","lightblue","red"),colNA="lightyellow",main="change under 2050rcp26",legend=F,xlab="Longitude(°)",ylab="Laitude(°)")
plot(RSB_5026$Diff.By.Pixel,col=c("blue","#49BE00","white","red"),colNA="#91522d",main="change under 2050rcp26",legend=F,xlab="Longitude(°)",ylab="Laitude(°)")
legend("bottomright",legend = c("loss","stable","gain","unsuitable"),fill=c("blue","#49BE00","red","white"),col=c("blue","#49BE00","red","#91522d"),bty="n")


plot(RSB_5085$Diff.By.Pixel,col=c("blue","green","lightblue","red"),colNA="lightyellow",main="change under 2050rcp85",legend=F,xlab="Longitude(°)",ylab="Laitude(°)")
plot(RSB_5085$Diff.By.Pixel,col=c("blue","#49BE00","white","red"),colNA="#91522d",main="change under 2050rcp85",legend=F,xlab="Longitude(°)",ylab="Laitude(°)")
legend("bottomright",legend = c("loss","stable","gain","unsuitable"),fill=c("blue","#49BE00","red","white"),col=c("blue","#49BE00","red","#91522d"),bty="n")


plot(RSB_0026$Diff.By.Pixel,col=c("blue","green","lightblue","red"),colNA="lightyellow",main="change under 2100rcp26",legend=F,xlab="Longitude(°)",ylab="Laitude(°)")
plot(RSB_0026$Diff.By.Pixel,col=c("blue","#49BE00","white","red"),colNA="#91522d",main="change under 2100rcp26",legend=F,xlab="Longitude(°)",ylab="Laitude(°)")
legend("bottomright",legend = c("loss","stable","gain","unsuitable"),fill=c("blue","#49BE00","red","white"),col=c("blue","#49BE00","red","#91522d"),bty="n")


plot(RSB_0085$Diff.By.Pixel,col=c("blue","green","lightblue","red"),colNA="lightyellow",main="change under 2100rcp85",legend=F,xlab="Longitude(°)",ylab="Laitude(°)")
plot(RSB_0085$Diff.By.Pixel,col=c("blue","#49BE00","white","red"),colNA="#91522d",main="change under
```

```
2100rcp85",legend=F,xlab="Longitude(° )",ylab="Laitude(° )")
legend("bottomright",legend =
c("loss","stable","gain","unsuitable"),fill=c("blue","green","red","lightblue"),col=c("blue","green","red","lightyellow"),bty="n")
legend("bottomright",legend =
c("loss","stable","gain","unsuitable"),fill=c("blue","#49BE00","red","white"),col=c("blue","#49BE00","red","#91522d"),bty="n")
```
