# Supplementary material for: Unveiling the suitable habitats and future conservation strategies of Tridacna maxima in the Indo‐Pacific core area based on species distribution model
Source: Ecol Evol. 2024 Sep 4;14(9):e70187. doi: 10.1002/ece3.70187 (PMC11372821; doi:10.1002/ece3.70187)
Supplement: Supplementary file 2 — Figure S1 [file ECE3-14-e70187-s002.docx]

**Supplementary material**

**
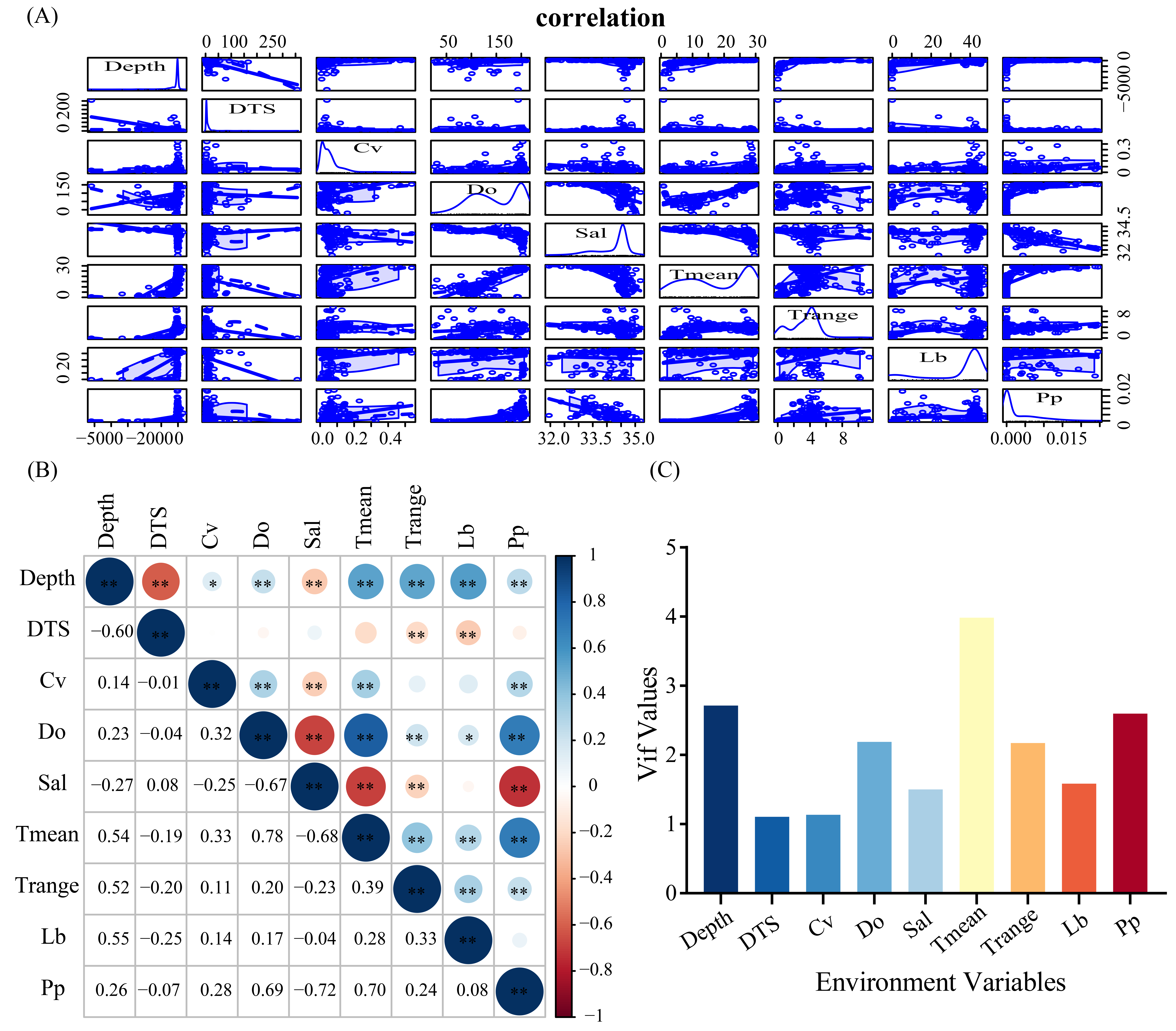
**

**Supplementary Figure 1** Results of collinearity analysis of nine predictors. Scatter collinearity analysis (A), Pearson’s correlation analysis (B), and variance inflation factor analysis (C) results for nine predictor variables. Depth - ocean depth; DTS – distance to shore; Cv - current velocity; Do - dissolved oxygen; Sal - salinity; Tmean – temperature mean; Trange – temperature mean; Lb - Light level at bottom; Pp - Phytoplankton.


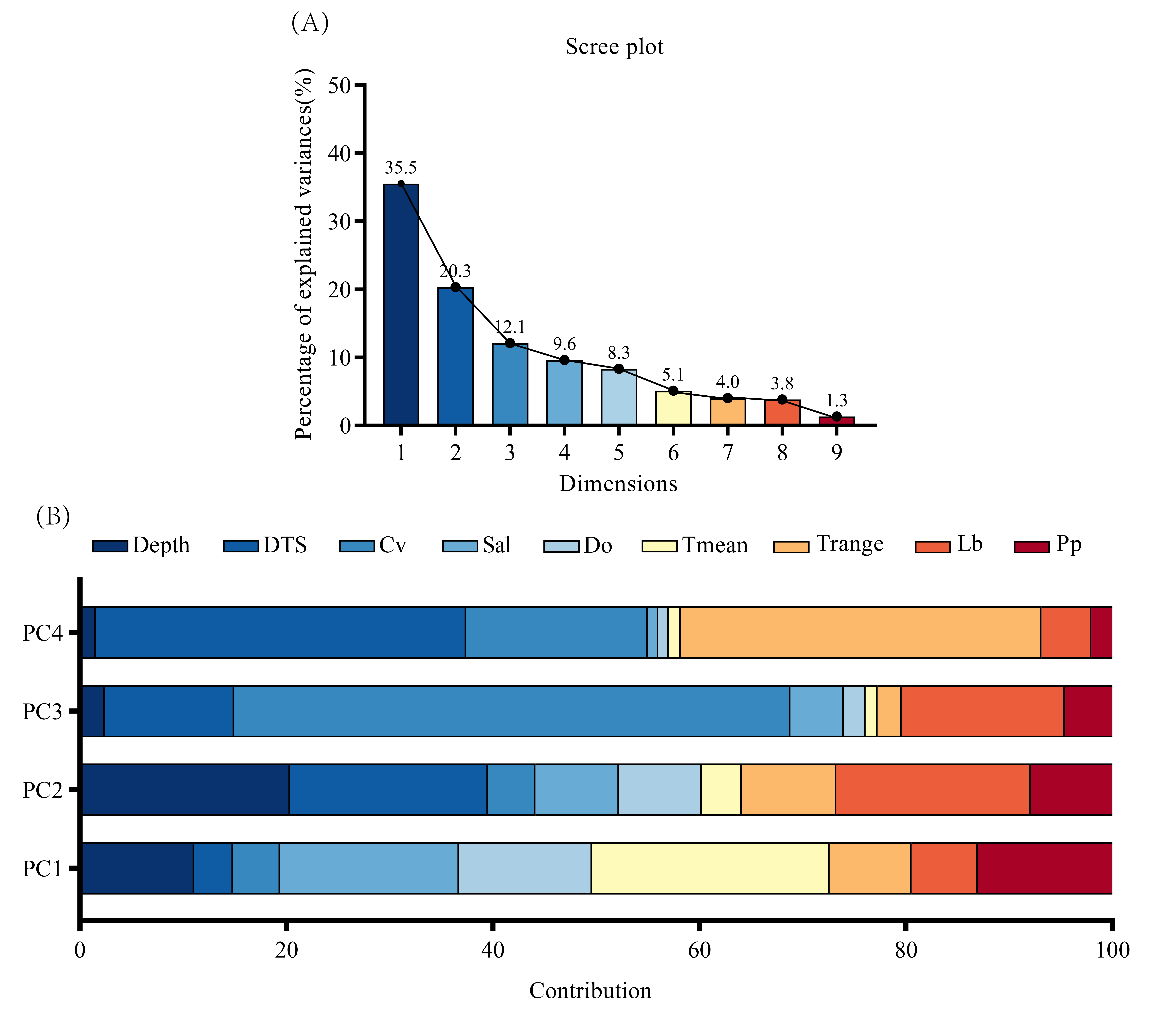


**Supplementary Figure 2** Ecological niche space differences. (A) Percentage of explained variance of each principal component of principal component analysis for the nine selected predictors. (B) Contribution of environmental predictors to each principal component (PC). Depth - ocean depth; DTS – distance to shore; Cv - current velocity; Do - dissolved oxygen; Sal - salinity; Tmean – temperature mean; Trange – temperature mean; Lb - Light level at bottom; Pp - Phytoplankton.





**Supplementary Figure 3** The response curves of *Tridacna maxima* occurrence probability against the two most important driving factors based on the species-level (A1, A2) and population -level model (including the Eastern Indian Ocean – South Sea population (B1, B2) and the Western Pacific – Indonesia population (C1, C2)). DTS – distance to shore; Tmean – temperature mean; Lb - Light level at bottom.

**Supplementary Table 1.** Total niche differentiation (βTotal) between populations and the proportion of niche shift and niche contraction/expansion. EIOS, East Indian Ocean-South China Sea; WPI, West Pacific-Indonesia.

| Populations Pair | βTotal | Niche shift | Niche Contraction/Expansion |
| --- | --- | --- | --- |
| EIOS-WPI | 0.61 | 0.28 | 0.33 |
